# *Euphorbia* species latex: A comprehensive review on phytochemistry and biological activities

**DOI:** 10.3389/fpls.2022.1008881

**Published:** 2022-10-06

**Authors:** Rania Benjamaa, Abdelkarim Moujanni, Neha Kaushik, Eun Ha Choi, Abdel Khalid Essamadi, Nagendra Kumar Kaushik

**Affiliations:** ^1^Laboratory of Biochemistry, Neurosciences, Natural Resources and Environment, Faculty of Sciences and Technologies, Hassan First University of Settat, Settat, Morocco; ^2^Department of Biotechnology, College of Engineering, The University of Suwon, Hwaseong-si, South Korea; ^3^Department of Electrical and Biological Physics, Plasma Bioscience Research Center, Kwangwoon University, Seoul, South Korea

**Keywords:** *Euphorbiaceae*, *Euphorbia* species, latex, chemical constituents, biological applications

## Abstract

The genus *Euphorbia* includes about 2,000 species commonly widespread in both temperate and tropical zones that contain poisonous milky juice fluid or latex. Many species have been used in traditional and complementary medicine for the treatment of various health issues such as dropsy, paralysis, deafness, wounds, warts on the skin, and amaurosis. The medicinal applications of these species have been attributed to the presence of various compounds, and most studies on *Euphorbia* species have focused on their latex. In this review, we summarize the current state of knowledge on chemical composition and biological activities of the latex from various species of the genus *Euphorbia.* Our aim was to explore the applications of latex extracts in the medical field and to evaluate their ethnopharmacological potential. The databases employed for data collection, are obtained through Web of Science, PubMed, Google Scholar, Science Direct and Scopus, from 1983 to 2022. The bibliographic data indicate that terpenoids are the most common secondary metabolites in the latex. Furthermore, the latex has interesting biological properties and pharmacological functions, including antibacterial, antioxidant, free radical scavenger, cytotoxic, tumor, anti-inflammatory, healing, hemostatic, anti-angiogenic, insecticidal, genotoxic, and mutagenic activities. However, the role of other components in the latex, such as phenolic compounds, alkaloids, saponins, and flavonoids, remains unknown, which limits the application of the latex. Future studies are required to optimize the therapeutic use of latex extracts.

## Introduction

Plant latex is produced by more than 20,000 species from around 40 families (Bauer et al., 2014). It is a fluid found in specialized cells called “laticifera” that are located throughout the plant (Ramos et al., 2020) and can have different colors: white, yellow, red, or colorless. Because of its sticky properties, the latex has been implicated in the defense against herbivorous insects and used to produce rubber (Agrawal and Konno, 2009). In addition, the latex of various plant species contains a wide variety of bioactive compounds, including proteins, enzymes, alkaloids, glycosides, cardenolides, terpenoids, furanocoumarins, and starch (Konno, 2011). Moreover, the water insoluble fraction of the latex from the families *Euphorbiaceae*, *Asclepiadaceae*, and *Caricaceae* has shown lipase activity and can be used as a useful biocatalyst for several synthetic applications in the food, pharmaceutical, and detergent industries (Paques and Macedo, 2006).

*Euphorbiaceae* is one of the largest and oldest plant families in the world, comprising approximately 300 genera and 8,000 species (Webster, 1987). This is one of the plant families with latex-producing species (Lewinsohn, 1991). The *Euphorbia* genus (commonly called spurge) incorporates a wide variety of plants with biological and medical applications (Kemboi et al., 2020). The species are distributed in both temperate and tropical regions (Pahlevani and Mozaffarian, 2011), with endemic species such as *E. resinifera* in Morocco (Chakir et al., 2016), *E. cubensis, E. helenae, E. munizii*, and *E. podocarpifolia* in Cuba (Steinmann et al., 2007), *E. polycaulis* in Iran (Nasr et al., 2018), *E. hainanensis* in China (Tian et al., 2018), *E. fauriei* and *E. garanbiensis* in Korea and Taiwan (Ki-Ryong, 2004), and *E. boetica* in the Iberian peninsula (Narbona et al., 2007). Plants in this genus contain a white acrid, poisonous milky juice fluid or latex that comes out when cut or damaged (Bigoniya and Rana, 2008) and is extremely irritating to the skin (Salehi et al., 2019).

The latex from several *Euphorbia* species has been chemically investigated. It contains different biological compounds, such as triterpenoids (Palocci et al., 2003; Kemboi et al., 2020) diterpenes, ingenol, 12-deoxyphorbol esters (Priya and Rao, 2011), triterpene alcohols, lanosterol,(Giner et al., 2000), fatty acids, proteins, and enzymes (Spanò et al., 2012). The terpenoids are the most abundant components of this genus, which are known to have pharmacological activities, which can offer a wide range of medicinal applications.

Furthermore, the latex of some *Euphorbia* species has been used in traditional medicine to treat wounds and warts on the skin (Özbilgin et al., 2019) as well as some nervous diseases, dropsy, paralysis, deafness, and amaurosis (Gewali et al., 1989).

To our knowledge, no literature review provides a comprehensive study on the latex of the genus Euphorbia. Here, we review the current state of knowledge on the ethnomedicinal uses, phytochemical composition, and biological activities of the latex from more than 20 species of *Euphorbia*. The main objective of this study is to present a database of knowledge and research trends on latex of the genus *Euphorbia* with the aim of providing basic data to promote future pharmacological and phytochemical studies on spurge latex.

## Distribution

The genus *Euphorbia* includes several species distributed in both temperate and tropical zones (El-Ghazaly and Chaudhary, 1993). However, many species are also present in non-tropical areas such as Africa and Central and South America (Liang et al., 2014). Certain species are distributed in India, specifically in the North and West (Pascal et al., 2017). This genus is represented in Taiwan by eight species (Lin and Hsieh, 1991). There are about 90 species mostly concentrated in Iran and 91 species in Turkey (Nasseh et al., 2018), with about 70 species found in China (Liang et al., 2014). On the other hand, in Brazil, the genus is represented by about 64 species, with a degree of endemism of about 50% (31 spp.) (Steinmann et al., 2007).

## Description

The genus contains several species, which can be annual or perennial, xerophytes, woody shrubs, or trees with a caustic and poisonous milky latex (Berg, 1990). They are characterized by the presence of fine or thick and fleshy or tuberous roots (Pascal et al., 2017). The fruits are basically fleshy, with explosive dehiscence (Dorsey, 2013). The species are generally recognized by their inflorescences, which are called cyathium and resemble a dicotyledonous flower. Each inflorescence contains a female flower surrounded by several male flowers and is composed of cup-like involucre formed by two bracts bearing four or five often horned glands (Prenner and Rudall, 2007).

## Phytochemical profile of *Euphorbia* latex

**FIGURE 1:**
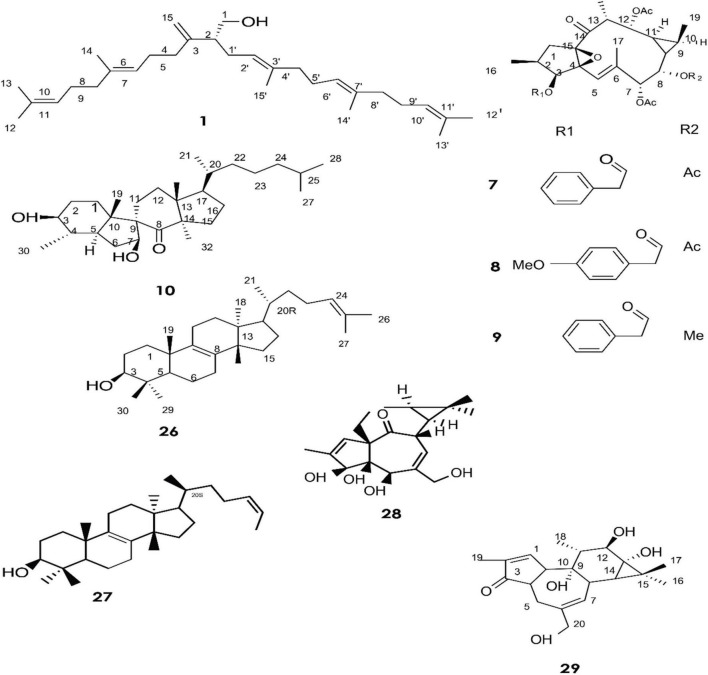
Structure of chemicals compound **1, 7-10, 26-29 (**Giner et al., 2000; Daoubi et al., 2007; de Souza et al., 2019).

**FIGURE 2:**
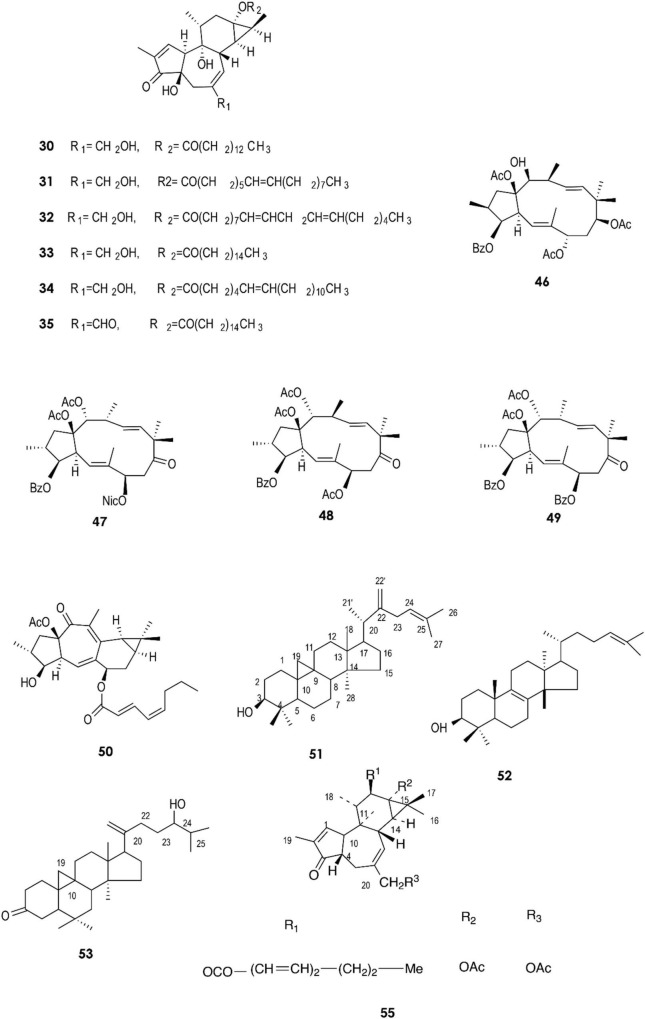
Structure of chemicals compounds **(30-35, 46-53)** and **(55)** (Urones et al., 1988; Ilyas et al., 1998; Mallavadhani et al., 2004; Hua et al., 2015; Deng et al., 2021).

**FIGURE 3:**
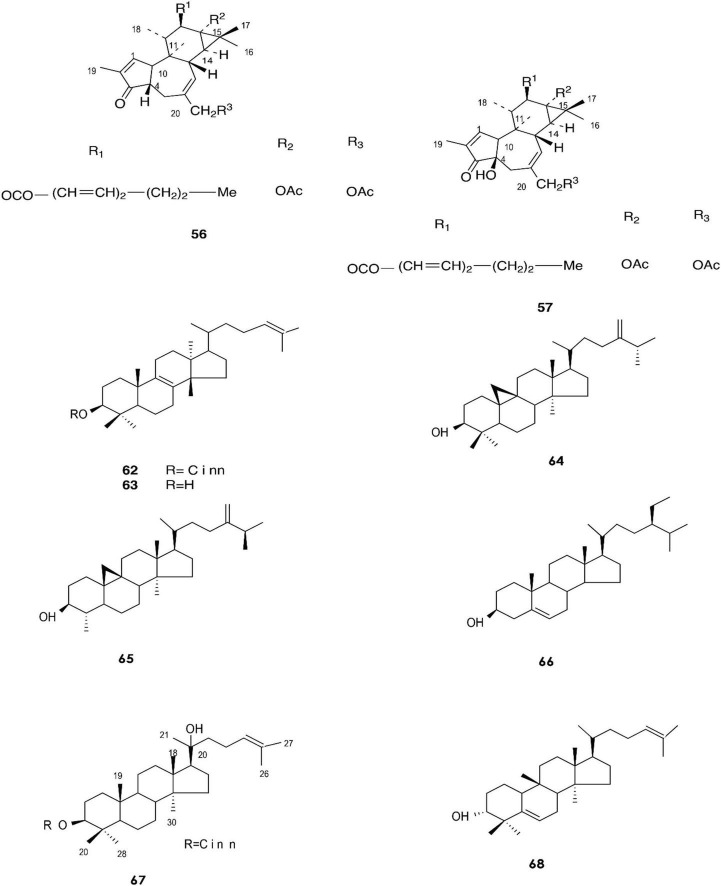
Structure of chemicals compounds **(56-57, 62-68)** (Urones et al., 1988; Gewali et al., 1990).

**FIGURE 4:**
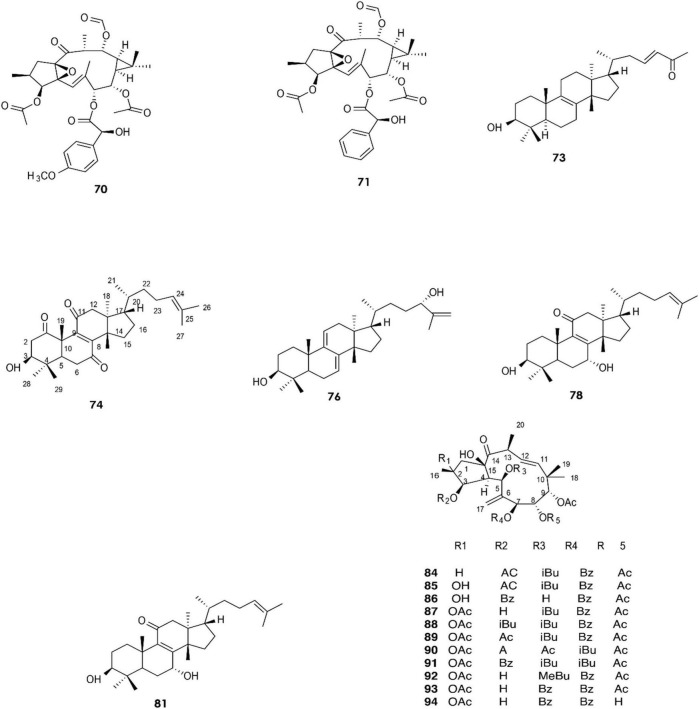
Structure of chemicals compounds **(70-74; 76; 78; 81) and (84-94)** (Esposito et al., 2016; Li et al., 2022).

**FIGURE 5:**
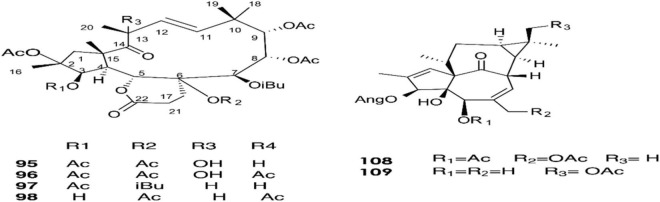
Structure of chemicals compounds (**95-98**) and (**108-109)** (Marco et al., 1999; Esposito et al., 2016).

**FIGURE 6:**
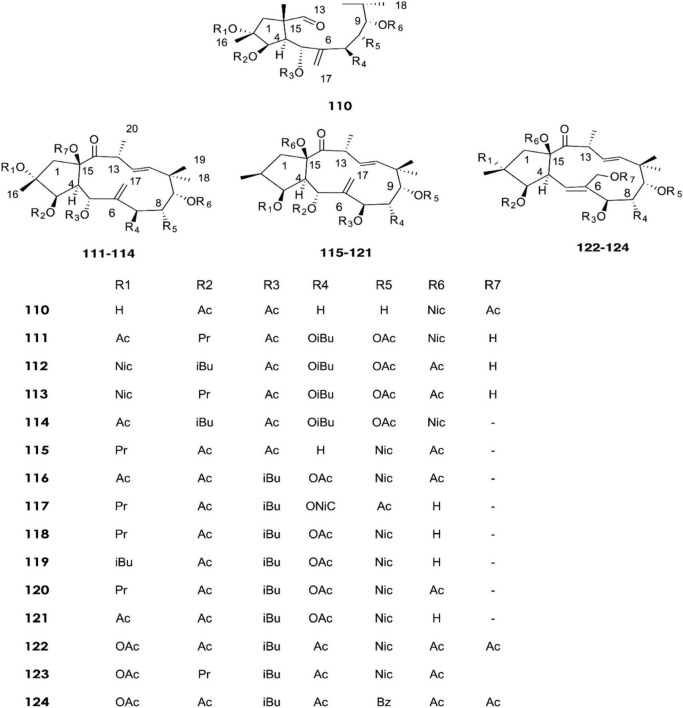
Structure of chemicals compounds (**110-124)** (Krstić et al., 2018).

Phytochemical investigations on different species of euphorbia have shown the presence of diversity of constituents, mainly terpenoids, enzymes and Natural Rubber. Table 1 shows the major terpenoids and Figures 1–6 showed the chemical structures of terpenoids isolated from different *Euphorbia* species.

**TABLE 1:** Chemical Constituents of euphorbia genus latex.

Species	Compounds	References
*E. peplus*	Peplusol (**1**) Obtusifoloio (**2**), lanosterol (**3**), 24-methylenelanosterol (**4**), cycloartenol (**5**) and 24-methylenecycloartano (**6**).	Giner et al., 2000
*E. officinarum*	7,8,12-triacetate 3-phenylacetate (**7**), ingol 7,8,12 triacetate 3-(4-methoxyphenyl)acetate (**8**), 8 methoxyingol 7,12-diacetate 3-phenylacetate (**9**), 3S,4S,5R,7S,9R,14R-3,7-dihydroxy-4,14-dimethyl-7[8 → 9] Abeo-cholestan-8-one (10), 3β-acetoxy-norlup-20-one (**11**) and 4α,14α-dimethyl-5α-cholest-8-ene (**12**).	Daoubi et al., 2007; Smaili et al., 2017
*E. obtusifolia*	(2R,3R,4R,5R,7S,8S,9S,11E,13S,15R)-2,3,5,7,8,9,15-Heptahydroxyjatropha-6(17),11-diene-14-one-7,8,9-triacetate-2,5-bis(2-methylbutyrate) (**13**), (2R,3R,4R,5R,7S,8S,9S,11E,13S,15R)-2,3,5,7,8,9,15-Heptahydroxyjatropha-6(17),11-diene-14-one-7,8,9-triacetate-2-isobutyrate-5-(2-methylbutyrate) (**14**), (2R,3R,4R,5R,7S,8S,9S,11E,13S,15R)-2,3,5,7,8,9,15-Heptahydroxyjatropha-6(17),11-diene-14-one-7,8,9-triacetate-2-nicotinate-5-(2-methylbutyrate) (**15**), (2R,3R,4R,5R,7S,8S,9S,11E,13S,15R)-2,3,5,7,8,9,15-Heptahydroxyjatropha-6(17),11-diene-14-one-8,9-diacetate-7-isobutyrate-2,5-bis(2-methylbutyrate) (**16**), (2R,3R,4R,5R,7S,8S,9S,11E,13S,15R)-2,3,5,7,8,9,15-Heptahydroxyjatropha-6(17),11-diene-14-one-2,8,9-triacetate-7-isobutyrate-5-(2-methylbutyrate) (**17**), (2R,3R,4R,5R,7S,8S,9S,11E,13S,15R)-2,3,5,7,8,9,15-Heptahydroxyjatropha-6(17),11-diene-14-one-7,9-diacetate-8-benzoate-2,3-bis(2-methylbutyrate) (**18**), (2R,3R,4R,5R,7S,8S,9S,11E,13S,15R)-2,3,5,7,8,9,15-Heptahydroxyjatropha-6(17),11-diene-14-one-8,9-diacetate-7-isobutyrate-2,3-bis(2-methylbutyrate*)* (**19**), 4,20*-Dideoxyphorbol* 12,13*-bis(isobutyrate)* ***(20)***, 4*-Deoxyphorbol 12,13-bis(isobutyrate)* ***(21)***, 17- *Acetoxy*-4-*deoxyphorbol* 12,13-*bis(isobutyrate)* (**22**) 17-*Acetoxy*-4,20-dideoxyphorbol *12,13-*bis(isobutyrate) ***(23****),4-*deoxyphorbol 12,13-bis(isobutyrate) 20-acetate (**24**) and 4-epi-4-deoxyphorbol ester: 4-Epi-4-deoxyphorbol 12,13-bis (isobutyrate) (**25**).	Marco et al., 1999
*E. tirucalli L*	Euphol (**26);** tirucallol (**27**), ingenol (**28**) and 4-desoxyphorbol (**29).**	de Souza et al., 2019
*E. fischeriana*	12-deoxyphorbol-13-tetradecanoate (**30**), 12-deoxyphorbol-13- (7Z)-hexadecenoate (**31**), 12-deoxyphorbol-13-(9Z, 12Z)-octadecadienoate (**32**), 12-deoxyphorbol-13-hexadecanoate (**33**), 12-deoxyphorbol-13-(6Z)- octadecenoate (**34**) and 12- deoxyphorbaldehy-13-hexadecanoate (**35).**	Deng et al., 2021
*E. bicolor*	Resiniferatoxin (**36**) and Abietic Acid (**37).**	Basu et al., 2019
*E. umbellate*	Lanosterol (**38**), cycloartenol (**39**), tirucallol (**40**), taraxasterol (**41**),lupeol (**42**), phorbol-12,13,20-triacetate (**43);** 4-β phorbol (**44);** and 3 desoxo-3,16-dihydroxy-12-desoxyphorbol 3,13,16,20-tetraacetate (**45).**	Cruz et al., 2020
*E. helioscopia*	7α, 9β, 15β-triacetoxy-3β-benzoyloxy-14β-hydroxyjatropha-5E, 11E-diene (**46**), euphoheliosnoid A (**47**), epieuphoscopin B (**48**), euphoscopin C (**49**), euphohelioscopin A (**50).**	Hua et al., 2015
*E. nerifolia*	9, 19-cyclolanost-22(22’), 24-diene-3β-ol (Neriifoliene) (**51**), 5α-eupha-8, 24-diaene-3β-ol (Euphol) (**52**), 9, 19-cyclolanost-20(21)-en-24-ol-3-one (Neriifolione) (**53**) and cycloartenol (**54).**	Ilyas et al., 1998; Mallavadhani et al., 2004
*E. broteri*	12-0-(2Z, 4E-octadienoyl)-4-deoxyphorbol-13 20-diacetate (**55**), 12-0-(2Z, 4E-octadienoyl)-phorbol-13, 20- diacetate (**56**), 20-acetyl-ingenol-3-decadienoate (**57**), 3-0-tetradecanoyl-ingenol (**58**), 20-0-tetradecanoyl-ingenol (**59**) and 5-0-tetradecanoyl-ingeol (**60).**	Urones et al., 1988
*E. lacteal*	Tirucallol (**61**)	Fernandez-Arche et al., 2010
*E. antiquorum*	euphol 3-0-cinnamate (**62**), euphol (**63**), 24-methylenecycloartanol (**64**), cycloeucalenol (**65**), β -Sitosterol (**66**); 3-0-cinnamoyl-20- hydroxy derivative of lanostane or euphane (antiquol A) (**67**), 3- epi-anhydrohtsomentof (antiquol B) (**68**), and 4-Acetoxyphenol (**69).**	Gewali et al., 1990
*E. resinifera*	(2’S)-ingol 3,8-diacetate-7-(2’-hydroxy-6’- methoxyphenyl) acetate (Euphoresin A) (**70**); (2’S)-ingol 3,8-diacetate-7-(2’-hydroxy-phenyl) acetate (Euphoresin B) (**71**), euphatexol A (**72**), euphatexol B (27-nor-3-hydroxy-25-oxo-eupha-8, 23-diene) (**73**), euphatexols C (3 β- hydroxyeupha-8,24-diene-1,7,11-trione) (74), euphatexol D ((24 R)-eupha-7,9,25- triene-3,24-diol) (**75**), euphatexol E (**76**), euphatexol F (3b,7a)-dihydroxyeupha-8,24-diene-11-one) (**77**), euphatexol G (3b,7a)-dihydroxy-24-methyleneeupha-8-ene-11-one) (**78**).3β-hydroxy-12α-methoxylanosta-7,9(11),24-triene (**79**), 3β-hydroxy-12α-methoxy-24-methylene-lanost-7,9(11)-dien (**80**), 3,7-dioxo-lanosta-8,24-diene (**81**), and 3,7-dioxo-24-methylene-lanost-8-ene (**82).** Resiniferatoxin (**83**)	Fattorusso et al., 2002; Qi et al., 2019; Wang et al., 2019; Li et al., 2021, 2022
*E. dendroides*	Euphodendroidins E (**84**), euphodendroidins F (**85**), Euphodendroidin J (2R,3R,4S,5R,7R,8R,9R,13S,15R)-8,9-Diacetoxy-2,5,15-trihydroxy-3,7-dibenzoyloxy-14-oxojatropha-6(17), 11Ediene (**86**), euphodendroidins A (**87**), Euphodendroidin K, (2R,3R,4S,5R,7R,8R,9R,13S,15R)-2,8,9-Triacetoxy-15-hydroxy-7-benzoyloxy-3,5-diisobutyroyloxy-14-oxojatropha-6(17),11E-diene (**88**), Euphodendroidin L (2R,3R,4S,5R,7R,8R,9R,13S,15R)-2,3,8,9-Tetracetoxy-15-hydroxy-7-benzoyloxy-5-isobutyroyloxy-14-oxojatropha-6(17),11E-diene (**89**), jatrophane ester (**90**), Euphodendroidin M, (2R,3R,4S,5R,7R,8R,9R,13S,15R)-2,8,9-Triacetoxy-15-hydroxy-3-benzoyloxy-5,7-diisobutyroyloxy-14-oxojatropha-6-(17),11E-diene (**91**), Euphodendroidins B (**92**), Euphodendroidin N, (2R,3R,4S,5R,7R,8R,9R,13S,15R)-2,8,9-Triacetoxy-3,15-dihydroxy-5,7-dibenzoyloxy-4-oxojatropha-6(17),11Ediene (**93**), (2R, 3R, 4S, 5R, 7R, 8R, 9R, 13S,- 15R)-2,9-diacetoxy-3, 8, 15-trihydroxy-5,7-dibenzoyloxy-14-oxojatropha-6(17), 11E-diene (euphodendroidins O) (**94**), 13α-hydroxyterracinolides G (**95**), 13α-hydroxyterracinolides B (**96**), terracinolides J (**97**) and C (**98).**	Esposito et al., 2016
*E.* acrurensis	*19-Hydroxyingol 3,12-diacetate 7,8-ditiglate* ***(99)***, *19-Hydroxyingol 3,12,19-triacetate 8-tiglate* ***(100)*,** *19-Hydroxyingol 12,19-diacetate 8-tiglate* ***(101)*,** *Ingol 3,8,12-triacetate 8-isovalerate* ***(102)*,** *ingol-3,8,12-triacetate-7-angelate* ***(103)*,** *Ingol 3,12-diacetate 7,8-ditiglate* ***(104)*,** *ingol-3,8,12-triacetate-7-tiglat* ***(105)*,** *8-O-methyl-ingol-3,12-diacetate-7-tiglate* ***(106)*,** *3,12-di-o-acethyl-8-o-tigloyli,gol* ***(107)*,** *ingenol 3-angelate 5,20- diacetate* ***(108)*** *and diester of 5-deoxyingenol* ***(109).***	Marco et al., 1999
*E. nicaeensis*	3b,5a,15b-triacetyloxy-2a-hydroxy-9a-nicotinyloxyjatropha-6 (17),11E-diene-14-one (**110**), 2a,5a,8a-triacetyloxy-15b-hydroxy-7b-isobutanoyloxy-9a-nicotinyloxy-3b-propanoyloxyjatropha-6 (17),11E-diene-14-one (**111**), 5a,8a,9a-triacetyloxy-15b-hydroxy-3b,7b-diisobutanoyloxy-2a-nicotinyloxyjatropha-6 (17),11Ediene-14-one (**112**), 5a,8a,9a-triacetyloxy-15b-hydroxy-7b-isobutanoyloxy-2a-nicotinyloxy-3b-propanoyloxyjatropha-6 (17),11E-diene-14-one (**113**), euphodendrophane O (**114**),5a,7 b,15b-triacetyloxy-9a-nicotinyloxy-3b-propanoyloxyjatropha-6 (17),11Ediene-14-one (**115**), 3b,5a,8a,15b-tetraacetyloxy-9anicotinyloxy-7b-isobutanoyloxyjatropha-6 (17),11E-diene-14-one (**116**), 5a,9adiacetyloxy-15b-hydroxy-7b-isobutanoyloxy-8a-nicotinyloxy-3bpropanoyloxyjatropha-6 (17),11E-diene-14-one (**117**), euphodendrophanes A (**118**), B(**119**), C (**120**), N (**121**), F (**122**), Q(**123**)and S (**124**) 3S,24S)-tirucall-7-ene-3,24,25-triol (**125**), (3S,24R)-tirucall-7-ene-3,24,25-triol (**126**) and inoterpene C (**127).**	Krstić et al., 2018, 2019
*E. hermentiana.*	3,12-O-diacetyl-7-O-benzoyl-8 methoxyingo l (**128**), 3,12-O-diacetyl-7-O-tigloyl-8- methoxyingol (**129**), 3,12-O-diacetyl-7-0-angeloyl-8-methoxyingol (**130**), 3,7,12-0-triacetyl-8-0-benzoyl-18-hydroxyingol (**131**), 3,7,12-O-triacetyl-8-O-benzoylingol (**132**), 3,7,12-O-triacetyl-8-0-tigloylingol (**133**), 3,7,8,12-O-tetraacetylingol (**134**), 3,7,8,12,18-O-pentaacetyl-18-hydroxyngol (**135**), 3,7,12,18-O-tetraacetyl-8-o-benzoyl-18-hydroxy-ingol (**136**),7-0-benzoyl-8-methoxy-12-0-acetylingol (**137**), 8-methoxy-12-O-acetylingol (**138**), 7-0-tigloyl-8-methoxy-12-0- acetylingol (**139**), 8-0-benzoyl-12-0-acetylingol (**140**), 12-O-acetylingol (**141**), 7,12-O-diacetyl-8-O-tigloylingol (**142**) and 8-0-tigloyl-l2-0-acetylingol (**143**)	Lin and Kinghorn, 1983
*E. Drupifera*	eupha- 8, 24-diene-3-ol (**144**) and 12-deoxyphorbol-20-propanoate (**145).**	Famuyiwa et al., 2014
*E. polygonifolia*	3β,17a,20S)-Dammara-12,24-dien-3-ol (Polygonifoliol) (**146**), (3β,20S)-Dammara-13(17),24-dien-3-ol (Isotirucallol) (**147**), Dammaradienol (**148**), Dammaradienol (**149**), Lupeol (**150**), Lanosterol (**151**), Butyrospermol (**152**), Tirucallenol (**153**), 24-Methylenelanosterol (**154**), Cycloartenol (**155**), Taraxasterol (**156**), β-Amyrin (**157**), 24-Methylenecycloartanol (**158**), Taraxasterol (**159**), α-Amyrin (**160**) and Multiflorenol (**161).**	Giner and Schroeder, 2015

## Terpenoids

Phytochemical screening has revealed that terpenes are the main constituents isolated from the latex of different species of the *Euphorbia* genus (Shi et al., 2008). Most of them are identified using high-performance liquid chromatography (HPLC) (Deng et al., 2021), chromatography-mass spectroscopy (GC-MS) (Cruz et al., 2020), NMR spectroscopic analysis (Esposito et al., 2016), and thin layer chromatography (Daoubi et al., 2007).

A total of approximately 161 compounds have been reported from 19 species: *E. peplus, E. officinarum, E. obtusifolia, E. tirucalli L* (Compounds 1, 7–10, 26–29) (Figure 1), *E. fischeriana, E. bicolor, E. umbellate, E. helioscopia, E. nerifolia, E. broteri, E. lacteal, E. antiquorum* (Compounds 30–35, 46–53, 55–57, 62–68) (Figures 2, 3), *E. resinifera, E. dendroides, E. acrurensis* (Compounds 70–74, 76, 78, 81, 84–94) (Figure 4), and (Compounds 95-98; 108-109) (Figure 5), *E. nicaeensis* (Compounds 110–124) (Figure 6), *E. hermentiana, E. Drupifera, E. polygonifolia*. Their resources from different *Euphorbia* species are shown in Table 1.

Giner et al. identified six triterpene alcohols from *E. peplus* latex **(1–6)** (Giner et al., 2000). Three new ingol diterpenes **(7–9)** and a novel spirotriterpene **(10)** were isolated from the dried latex of *E. officinarum* collected from Morocco. Theirs structures were elucidated by means of mass spectrometry, extensive 1D and 2D NMR (COSY, HMQC, HMBC, and NOESY), and X-ray analysis (Daoubi et al., 2007).

Other compounds have also been confirmed from the latex of *E. officinarum*, including **(11)** and **(12)**. These were identified on the basis of spectroscopic data (NMR), which showed a singlet at δ 2.15 ppm assigned to the methyl of the carbonyl group at C-20 for compound **(12)** and a doublet of doublet at δ 3.41 due to the resonance of H-3 for product **(13)** (Smaili et al., 2017). Furthermore, twelve new compounds (**13–25**) were isolated from *E. obtusifolia* latex (Marco et al., 1999). Phytochemical characterization of Brazilian *E. tirucalli* latex resulted in the isolation of triterpenes such as **(26)** and **(27)** using Fourier transform-ion cyclotron resonance mass spectrometry (FT-ICR MS) and Atmospheric Pressure Chemical Ionization APCI (+) FT-ICR MS. In addition, two diterpene esters **(28, 29)** were isolated by electrospray ionization Fourier transform ion cyclotron mass spectrometry ESI (-) FT-ICR MS and ESI (-) FT-ICR MS/MS (de Souza et al., 2019).

The fresh latex collected from the roots of *E. fischeriana* has been analyzed using spectroscopic methods, HPLC, and GC-MS analyses. The diterpenoids profile contained six aliphatic tigliane diterpenoids **(30-35)** that were identified as major compounds.

**FIGURE 7:**
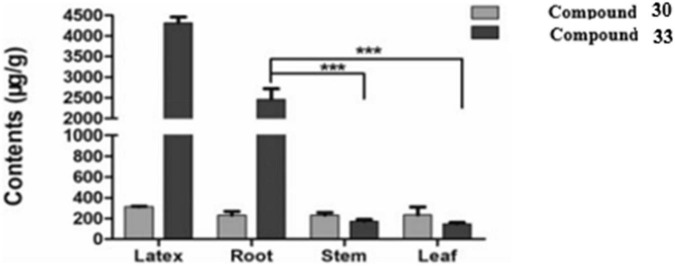
Content of compounds **30** and **33** in the different parts of *E. fischeriana* (***p < 0.001, student’s test) (Deng et al., 2021).

Quantitative analyses by High-Performance Liquid Chromatography with Diode-Array Detection (HPLC-DAD) revealed that compounds **(30)** and **(33)** were also present in the roots, stems, and leaves of *E. fischeriana* at varying proportions. On the other hand, **(30)** and (**33**) were mainly accumulated in the latex, with a value of greater than 232.31 ± 35.96 μg/g and 4,319.07 ± 143.26l μg/g, respectively (Figure 7). These two diterpenoids exhibited a marked antifeedant activity against *Helicoverpa armigera*, with EC50 values of 2.59 and 15.32 μg/cm^2^, respectively (Deng et al., 2021).

Analysis by UPLC-ESI-MS/MS of latex methanolic extract samples from ***E. bicolor*** collected in Denton County, TX, USA identified two diterpenes **(36)** and **(37)** (Basu et al., 2019) responsible for the anti-inflammatory (Fernandez et al., 2001) and analgesic activity, respectively.

Compounds **(38)** to **(43)** were isolated from the hexane fraction of the latex from *E. umbellata*. On the other hand, the diterpenes **(44)** and **(45)**, which were isolated from dichloromethane and ethanol fractions, are characterized by a tigliane nucleus (Cruz et al., 2020). In 2015, a new jatrophane diterpenoid, **(48)**, and four known macrocyclic diterpenoids, **(46), (47), (49)**, and **(50)**, were isolated from the stem latex of *E. helioscopia* using reversed-phase HPLC equipped with a diode array detector and recorded at 238 nm. It was observed that **(50)** moderately inhibits the release of the cytokines TNF-α (IC_50_ = 23.7 ± 1.7 μM) and IL-6 (IC_50_ = 46.1 ± 1.1 μM) and the chemokine MCP-1 (IC_50_ = 33.7 ± 3.8 μM) by lipopolysaccharide (LPS)-induced RAW 264.7 macrophages (Hua et al., 2015). Compounds **(51)** and **(52)** have been reported in *E. neriifolia* (Mallavadhani et al., 2004); compounds **(53)** and (**54**) were isolated from the same species and their structures were identified using chemical and physical data (1H NMR, 13C NMR, IR, and mass spectra) (Ilyas et al., 1998).

Several studies have reported that many terpenoids from the latex of *Euphorbia* species possess biological activities. Compound (61) constitutes 0.3% of the latex obtained by incision from the leaves of *E. lacteal*. It was identified by comparing the spectroscopic data (NMR and CG-MS) from the n-hexane/ethyl ether fraction and has been suggested to exhibit an anti-inflammatory activity, as it suppresses ear edema in a mouse model and inhibits nitrite production at a concentration of 100 mM in lipopolysaccharide-stimulated mouse macrophages (Fernandez et al., 2001).

Gewali et al. reported the isolation of compounds **(62–69)** in *E. antiquorum* latex (Gewali et al., 1990).

In 2019, the two diterpenes **(70)** and **(71)** were isolated from a methanol extract of the latex of *E. resinifera Berg*, and their structures were elucidated by HR-ESI-MS, IR, UV, 1D, and 2D NMR (Wang et al., 2019). Moreover, diterpenoid **(83)** was isolated by Fattorusso et al. (2002). Twelve compounds **(70–82)** have been identified from *E. resinifera*. Two triterpenoids were isolated by Qi et al., compound **(72)**, which was reported for the first time and was shown to contain a tetrahydrofuran ring, and **(73)** (Qi et al., 2019). Furthermore, five triterpenoids **(74–78)** were discovered by Li et al. (2022), and **(79–82)** were isolated in 2021 (Li et al., 2021).

The latex of *E. dendroides* was studied for its chemical composition and anti- Chikungunya virus (CHIKV) activities. The results showed the presence of six new jatrophane esters, **(86), (88), (89), (91), (93)**, and **(94)**, and nine known compounds, **(84)**, **(85)**, **(87)**, **(92)**, **(90)**, **(95)**, **(96)**, **(97)**, and **(98)**.

In an evaluation of 15 compounds, **(90)** and **(97)** showed anti-CHIKV activity with EC50 values of 5.5 ± 1.7 and 15.0 ± 3.8 μM, respectively (Esposito et al., 2016).

Marco et al. reported in 1998 nine ingol esters **(99–107)** bearing various types of acyl groups, acetyl and tigloyl moieties, and two known ingenol esters as minor compounds in the latex of *E. acrurensis*. The structure of compounds **(99) and (104)** is characterized by the presence of two tiglate esters in C-7 and C-8. Compound (**105**) is characterized by the presence of tiglate at C-7. In contrast, compound **(103)** has an angelate residue (Marco et al., 1999).

In recent years, fifteen diterpenoids **(110–124)** were extracted from the latex of *E. nicaeensis* samples collected in Serbia (Krstić et al., 2018). Meanwhile, three tetracyclic triterpenes **(125–127)** were isolated in 2019 (Krstić et al., 2019).

Four new ingol esters **(128–131)** and compounds **(132–143)** were isolated from *E. hermentiana* latex (Lin and Kinghorn, 1983). Compounds **(144)** and **(145)** were obtained from methylated spirit extract of the *E. drupifera* latex by Famuyiwa et al., and their structures were determined by 1D-NMR and MS (Famuyiwa et al., 2014). In 2015, 16 triterpene alcohols **(146–161)** were identified by Giner et al. from *E. polygonifolia* latex (Giner and Schroeder, 2015).

## Enzymes

**FIGURE 8:**
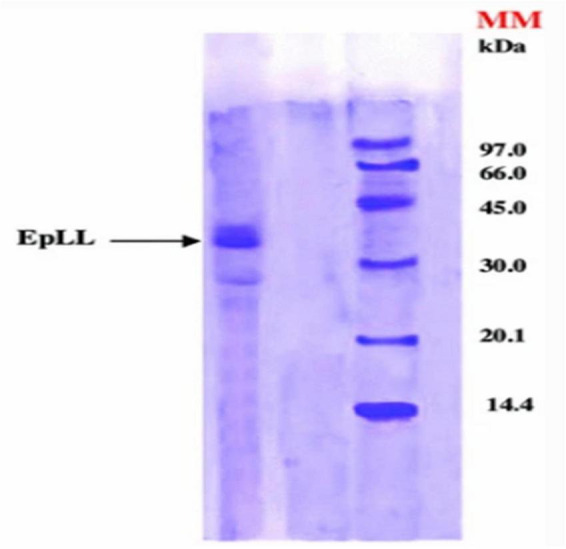
The molecular weight of the lipase from *E. peplus* latex (EPLL) obtained by Sodium dodecyl sulfate–polyacrylamide gel electrophoresis (SDS-PAGE) (Lazreg Aref et al., 2014).

Screening of *Euphorbia* latex has revealed the presence of many enzymes, including proteolytic enzymes that may be involved in plant defense against certain pathogens and external environmental conditions (Domsalla et al., 2010; Fais et al., 2021). The catalytic properties of lipases contained in the latex of *E. unispina* have been described by Mazou et al. (2017). The optimum temperature and pH for the hydrolytic activity of the lipases were 50°C and 5, respectively. The lipase was able to catalyze the hydrolysis of different purified Tunisian *E. peplus* triacylglycerols such as tripalmitin, trimyristin, trilaurin, tristearin, triolein, and trilinolein. In the same way, Lazreg Aref et al. studied the lipolytic activity of the latex lipase. The optimum lipase activity was obtained at 40°C and pH 8, with a molecular weight of about 40 kDa, which was determined using electrophoresis on dodecyl gel sodium sulfate (electrophoresis on gel (SDS-PAGE) (Figure 8). Tributyrin (TC4) and olive oil were used as substrates to determine the specific activity of the lipase, which was found to be 249 ± 12.45 and 161.4 ± 8.07 U/mg for TC4 and olive oil, respectively. However, the lipase activity was inhibited by sodium dodecyl sulfate (Lazreg Aref et al., 2014). Moreover, the biological properties of the proteases have been reported. A serine protease with a molecular weight of 61 KDa designated as EuRP-61 was well purified from *E. resinifera* latex and characterized. The enzyme was found to have a wide pH stability range of 1–14 and a denaturation tolerance of up to 65–66°C. The fibrinogenolytic activity of EuRP-61 was investigated, and the optimal degradation of fibrinogen was found to have a Michaelis constant (Km) of 4.95 ± 0.1 μM, a maximum velocity (Vmax) of 578.1 ± 11.81 ng min^–1^, and a catalytic efficiency (Vmax/Km) of 116.8 ± 1 ng μM^–1^ min^–1^ (Siritapetawee et al., 2020b). Siritapetawee et al. also studied the anticoagulant and antithrombotic activities of EuRP-61, and reported that this enzyme can hydrolyze human fibrin and inhibit platelet aggregation via the ADP receptor pathway (Siritapetawee et al., 2020a). Proteases such as euphorbain 1, eumiliin, mauritanicain, EuP-82, miliin, and euphorbams y-l, –2, and –3 have been purified and characterized from *E. lathyris, E. milii var.* hislopii, *E. mauritanica* L, *E. cf. lacteal, E*. *milii*, and *E. cyparissias*, respectively. The proteolytic activity of euphorbain1 is inhibited by diisopropyl fluorophosphates, the fibrinogenolytic activity of eumiliin is inhibited by β-mercaptoethanol and leupeptin. The mauritanicain is reduced in its proteolytic activity by aprotinin and AEBSF-HCl [4-(2-Aminoethyl)benzenesulfonylfluoride] and EuP-82 is inhibited by serine protease specific inhibitor phenylmethylsulfonyl fluoride (PMSF) (Lynn and Clevette-Radford, 1983, 1985; Fonseca et al., 2010; Moro et al., 2013; Siritapetawee et al., 2015; Flemmig et al., 2017). Furthermore, a protease has been isolated from *E. amygdaloides* latex using collapse of (NH4)_2_SO_4_ fractionation and ion-exchange chromatography. Maximum protease activity was observed at 60°C and pH 5 (Demir et al., 2005). Badgujar et al. found a clotting cysteine protease called Nivulia-II, which they purified from *E. nivulia Buch.-Ham* latex with DFPPNTCCCICC as the N-terminal amino acid sequence; the enzyme is characterized by a molecular weight of 43,670.846 Da and has an optimal activity at pH 6.3 and 50°C, which can be inhibited by common thiol blocking reagents (Badgujar and Mahajan, 2014).

Four enzymes have been purified from *E. characias* latex, an amine oxidase, a nucleotide pyrophosphatase/phosphodiesterase, a peroxidase, and a purple acid phosphatase, with molecular masses of 74, 5, 47, and 30 ± 10 kDa, respectively (Padiglia et al., 1998; Mura et al., 2008; Medda et al., 2011; Pintus et al., 2011). The serine protease purified from *E. hirta* has fibrinolytic, esterase, amidase, azocaseinolytic, fibrinogenolytic, and gelatinolytic activities. Enzyme activity was found to be inhibited by PMSF and AEBSF, and the N-terminal sequence was determined to be YAVYIGLILETAA/NNE (Patel et al., 2012). In addition, a class III endochitinase with important roles in cellular defense has been isolated from the latex of *E. characias*. This enzyme shows strong activity at 50°C and pH 5.0, and its chitinase activity can be enhanced by calcium and magnesium ions. Moreover, the enzyme was found to hydrolyze colloidal chitin, yielding N-acetyl-d glucosamine, chitobiose, and ketotriose as products (Spano et al., 2015).

## Natural rubber

**FIGURE 9:**
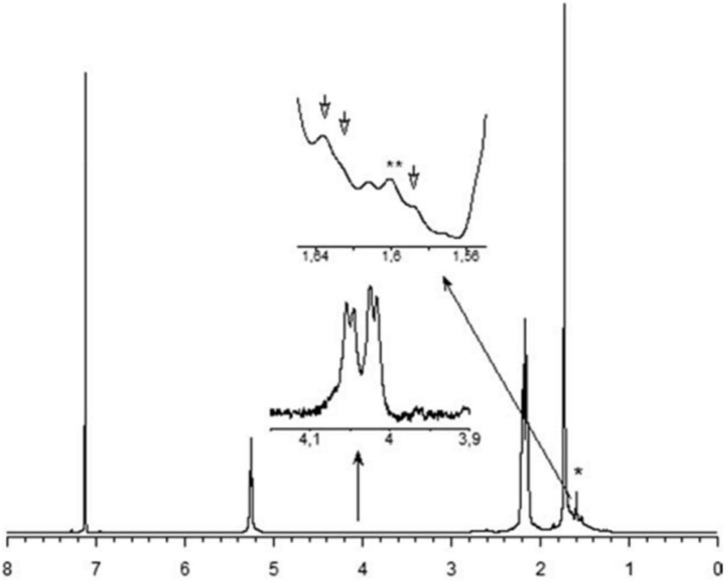
The ^1^ H NMR spectrum of rubber extracted from *E. characias* latex. Peaks at 5.31, 2.17, and 1.73 ppm are attributed to the olefinic, methylene and methyl protons, respectively, of the cis-1,4-polyisoprene (Spanò et al., 2012). * and ** indicates 1 H NMR residual signal of cyclohexane (1.43 ppm) and methyl-protons of a trans-isoprene unit (1.62 ppm), respectively.

**FIGURE 10:**
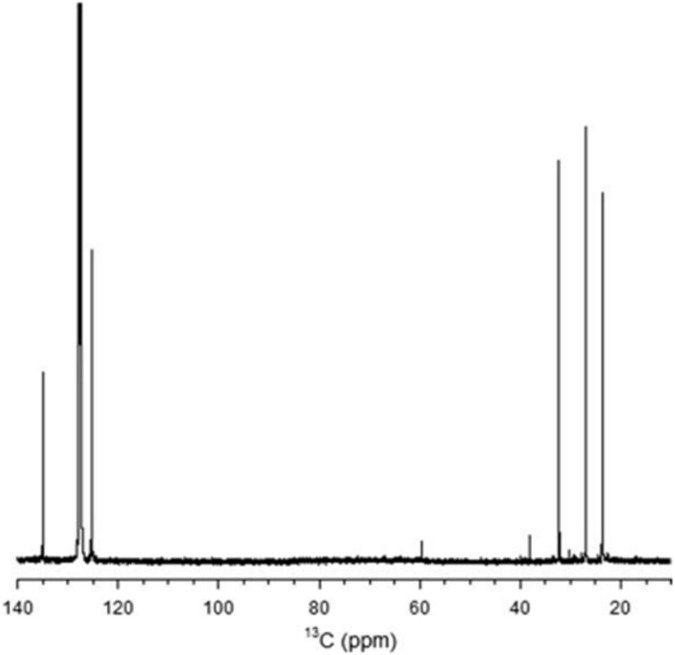
The ^13^C NMR spectrum of rubber extracted from *E. characias* latex. The peaks at 135.2,125, 32.2, 26.4, and 23.4 arises from the two ethylenic, two methylenic, and the methyl carbon atoms of the cis-1,4-polyisoprene, respectively (Spanò et al., 2012).

**FIGURE 11:**
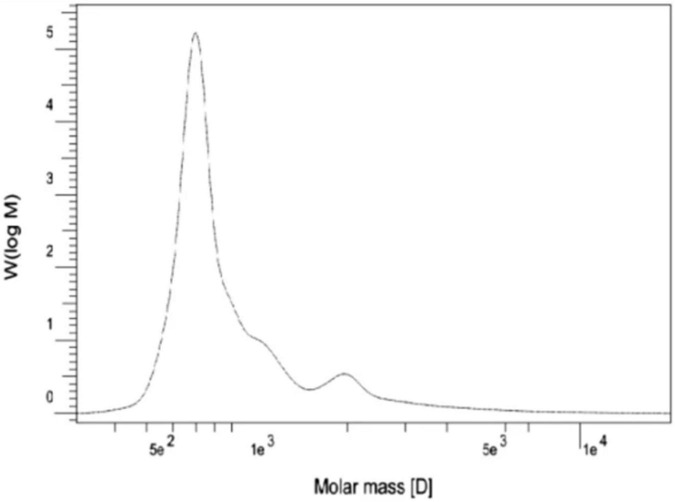
Molecular weight distribution of *E. macroclada* extracted rubber by GPC.

Natural rubber (NR) is an important polymer found in about 2,000 plant species (Arreguín, 1958). To date, *Hevea brasiliensis* is considered the most important rubber-producing plant (Laibach et al., 2015). NR from *E. characias* latex has been extracted using different solvents such as acetone, acetic acid, trichloroacetic acid, and Triton X-100, followed by successive treatments with cyclohexane/ethanol and characterized. Acetic acid has proven to be the most suitable solvent for rubber extraction, with yields of 14.3%. ^1^ H NMR, and ^13^C NMR analysis showed that the NR has a molecular weight of 93,000 Da and contains cis-1,4-polyisoprene as shown in Figures 9, 10 (Spanò et al., 2012). FT-IR, NMR, and GPC analyses also revealed that the NR from *E. macroclada* latex contains cis-1,4-polyisoprene, with a molecular weight of 8.180E+2 with polydispersity of 1.287 as shown in Figure 11 (Khan and Akhtar, 2003; Azadi et al., 2020) separated and characterized rubber hydrocarbon from *E. caducifolia* by different chemical methods. The analysis revealed a molecular weight of 15,275–88,405 (M), iodine value of hydrocarbon of 310.91–350.80%, percentage of unsaturation of 83.40–94.10%, a refractive index of 1.49200–1.49325, and a specific gravity of 0.93102– 0.93628, and identified cis-1,4-polyisoprene (Khan and Akhtar, 2003).

## Biological activities of *Euphorbia* latex

Several researchers have studied the biological activity of spurge latex extracts and their chemical constituents, both in vitro and in vivo. *Euphorbia* latex has antibacterial, antioxidant, anti-inflammatory, anti-angiogenic, wound healing, cytotoxic, hemostatic, genotoxic/mutagenic, and insecticidal activities. Supplementary Table 1 summarizes the results of various investigations concerning the biological activities of latex from some species of the genus *Euphorbia*.

## Antimicrobial activity

Several studies have explored the antibacterial activity of the latex from *Euphorbia* species (Supplementary Table 1). Most species in this genus exhibit moderate to strong antibacterial characteristics.

The agar well diffusion, disk diffusion, and broth microdilution methods have been applied in vitro to test the antimicrobial activity of fresh, diluted latex and some fractions isolated from latex by calculating the inhibition zone diameter and minimum inhibitory concentration (MIC). In addition, different solvents have been used to test the antimicrobial activity of latex or extracts against the Gram-positive bacteria *Bacillus pumilus*, *Staphylococcus aureus*, *Streptococcus pneumoniae*, *Bacillus subtilis*, and *Micrococcus luteus*, the Gram-negative bacteria *Escherichia coli*, *Citrobacter freundii*, *Klebsiella pneumoniae*, *Shigella flexneri, Proteus vulgaris, Pseudomonas aeruginosa, Salmonella typhi, Agrobacterium tumefaciens, Erwinia amylovora, P. syringae* pv. *tabaci*, and *Pseudomonas syringae* pv*. syringae*, and the fungal pathogens *Verticillium dahlia*, *Fusarium oxysporum* f. sp. *melonis*, and *Penicillium expansum*. In general, the fresh latex of *E. hirta* shows a promising activity against *B. pumilus* (24.98 mm), *S. aureus* (25.38 mm), *S. pneumoniae* (23.72 mm), *E. coli* (27.93 mm), *C. freundii* (23.54 mm), and *K. pneumoniae* (21.93 mm). Most of these recorded zones of inhibition are larger than those of the positive controls (vancomycin (22.29 mm), ceftriaxone (22.50 mm), ceftriaxone (22.50 mm), ciprofloxacin (22.36 mm), and levofloxacin (21.70 mm)(Hussain et al., 2014). The methanolic extract of latex from *E. antiquorum* displays moderate inhibitory effects against *E. coli* and *S. flexneri*, with inhibition zones of 5 and 4 mm respectively, but not against *K. pneumonia*, *S. aureus*, or *B. subtilis.* Using the agar plug method, researchers have shown that the methanolic extract of latex from *E. antiquorum* reduces the growth of *A. fumigatus*, *C. albicans*, and *A. flavus*, with inhibition zones of 12, 10, and 5–6 mm, respectively (Sumathi et al., 2011). ML et al. examined the antimicrobial activity of different solvent extracts (acetone, chloroform, and diethyl ether) of *E. heterophylla* latex. The acetone extract demonstrated a high zone of inhibition against most microbes, including *S. aureus*, *P. aeruginosa*, *B. subtilis*, *A. niger*, and *F. oxysporum*. The diethyl ether latex extract was more effective at inhibiting *P. vulgaris* and *Penicillium sp*.

In addition, the antimicrobial activity of the triterpene derivatives, 3β-acetoxy-norlup-20-one and 3-chloro-4α,14α-dimethyl-5α-cholest-8-ene, isolated from *E. officinarum* latex, has been determined. When used at concentrations of 100 and 200 μg/ml, they were shown to reduce conidia formation in six strains of *V. dahliae* (from 39 to 69%) as well as in *P. expansum* and *F. oxysporum* f. sp. *melonis* (from 70 to 96%). Moreover, they were also shown to inhibit the germination of all strains at concentrations of 2, 10, 100, and 200 μg/ml (ML et al., 2020).

The antibacterial activity of 3-chloro-4α, 14α-dimethyl-5α-cholest-8-ene has been demonstrated against *P. syringae* pv. *tabaci*, which causes tobacco wildfire disease, with an inhibition diameter of about 16 mm (Smaili et al., 2017). There are also reports of the antimicrobial activity of compounds other than triterpenes. For example, methyl palmitate, 5,9-hepta decadienoate, methyl 11 octadecenoate, methyl octadecenoate, and 3,7,11,15-tetramethyl-2-hexadecen-l-ol were isolated from *E. caducifolia*, and their antimicrobial activity was determined for a broad range of Gram-positive bacteria such as *S. aureus* (MIC = 262 μg/ml), *M. luteus* (MIC = 212 μg/ml), and *B. subtilis* (MIC = 187 μg/ml), Gram-negative bacteria such as *E. coli* (MIC = 225 μg/ml) and *S. typhi* (MIC = 275 μg/ml), and fungi such as *A. niger* (MIC = 150 μg/ml) and *C. albicans* (MIC = 175 μg/ml) (Goyal et al., 1970).

## Antioxidant activities and free radical scavenger activity

Numerous studies have reported the antioxidant effects of *Euphorbia* latex. Phenolic compounds and secondary metabolites are generally responsible for the antioxidant properties (Koh et al., 2002). The antioxidant action of latex from *E. dendroides L.* collected in Texas, USA was studied using different in vitro assays such as 2,2-diphenyl-2-picrylhydrazyl (DPPH), Trolox equivalent antioxidant capacity (TEAC), and Ferric reducing antioxidant power (FRAP) and a concentration range of 0.625–10 μg/mL. The DPPH, FRAP, and TEAC IC_50_ antioxidant activities were 2,927.01 ± 98.03, 4,383.13 ± 95.30, and 7,580.95 ± 97.65 μmols of trolox equivalents (TE)/100 g fresh weight of sample, respectively. This antioxidant power can be attributed to the polyphenols, specifically phenolic acids, and terpenoids contained in the latex of *E. dendroids* (Smeriglio et al., 2019). Abdel-Aty et al. have reported that the antioxidant properties of *E. tirucalli* latex extracts can be attributed to phenolic and flavonoid compounds. They found that the amounts of flavonoids and phenols found in the *E. tirucalli* latex extracts are about 4.3 and 10.5 mg EC/g latex, respectively. These were able to scavenge free radicals from DPPH and ABTS, with IC_50_ of 6.0 and 2.0 μg GAE/ml, respectively. In addition, the phosphomolybdate assay revealed that the latex also has a high reduction capacity, with an EC_50_ value of 6.5 μg/m (Abdel-Aty et al., 2019). The latex extract of *E. bicolor* samples collected in Texas, USA showed a dose-dependent ABTS radical of 80%, a DPPH scavenging effect of 8%, and a H_2_O_2_ radical of 30% at the concentration of 20–100 μg/mL. Moreover, the 2,2’-azino-bis(3-ethylbenzothiazoline-6-sulphonic acid) (ABTS) radical scavenging activity of the latex extract from *E. bicolor* is strongly correlated with the concentration of flavonoids and proanthocyanidins. The DPPH and NO radical scavenging activities of the extract show strong correlation with phenolic compounds and terpenoids contents. On the other hand, the H_2_O_2_ radical scavenging activity shows weak correlations with polyphenols contents (Basu et al., 2019).

## Insecticidal activity

The use of drugs to control parasites poses many challenges, such as the resistance to insecticides developed by the parasites and the environmental damage caused by the drugs (O’Brien, 1999; Daborn and Le Goff, 2004). The insecticidal activity of *E. bupleuroides* latex samples from the east of Algeria has been evaluated against German cockroach (*Blattella germanica***).** The insecticidal activity against adults and larvae was dependent on the concentration and time of exposure and found to be particularly effective against males and caterpillars (Azoui et al., 2016). The insecticidal activity of xylene-latex extracts from *E. antiquorum* collected in dry, intermediate, and wet zones of Sri Lanka has been studied against six species of insect pests: *Myzus persicae*, *Aphis gossypii*, *Aphis craccivora*, *brown planthopper* (*Nilaparvata lugens*), *paddybug (Leptocorisa oratorius*), and *blackbug (Scotinophara lurida*) (De Silva et al., 2008). The activity against two species of predatory ladybird beetles, *Harmonia octomaculata* and *Menochilus sexmaculatus* (*Cheilomenes sexmaculatus*), and the predatory spider *Lycosa pseudoannulata*, was determined using the Potters’ sprayer method. The three aphid species, *A. craccivora*, *A. gossypii*, and *M. persicae*, showed a high level of mortality toward the xylene-latex extract. On the other hand, *H. octomaculata* and *M. sexmaculatus* did not show any mortality for the xylene extract.

## Anti-inflammatory activity

**FIGURE 12:**
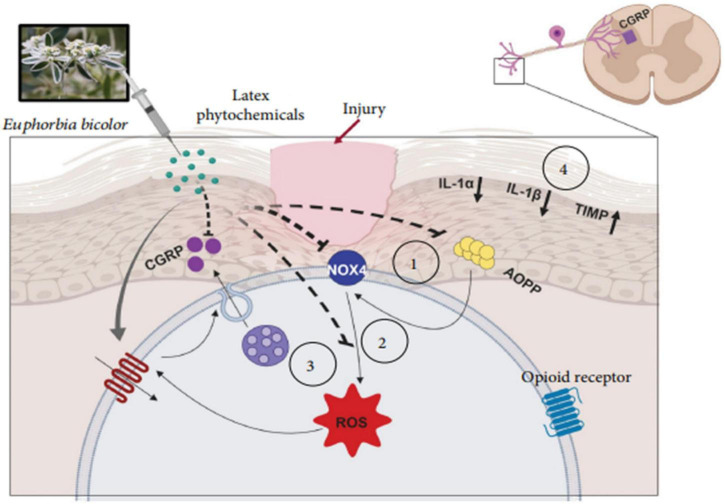
Proposed model of the mechanisms involved in *E. bicolor* latex extract-evoked peripheral, nonopioid analgesia (Basu et al., 2019).

The latex of the *Euphorbia* genus also has anti-inflammatory effects. The anti-inflammatory effect of a hydrosoluble fraction of *E. royleana* latex was investigated using different acute and chronic test models in rats and mice, with acetylsalicylic acid (ASA) as a positive control. The latex showed a significant dose-dependent anti-inflammatory activity, as evidenced by the reduction in the volume of exudate that resulted from the migration of leukocytes, and showed a weak inhibitory effect on the formation of granulomas induced by cotton pellets (Bani et al., 2000). The anti-inflammatory effects of *E. helioscopia* latex on carrageenan-induced paw edema have been tested in mice. The latex (200 mg/kg) showed maximal anti-inflammatory (68.75%) compared to the control (2 mg/kg indomethacin) (59.38%) (Saleem et al., 2015b). Moreover, Basu, P et al. showed that *E. bicolor* latex extracts induce analgesia by reducing the levels of oxidative stress biomarkers and pro-inflammatory cytokines/chemokines in a rat model of orofacial pain. Figure 12 shows a proposed model of the non-opioid mechanism that contributes to the peripheral analgesia induced by *E. bicolor* latex extracts. It shows that local injection of phytochemicals from *E. bicolor* latex at the site of injury may be effective in reducing oxidative stress by reducing the plasma levels of advanced oxidation protein products (AOPP) and increasing the expression of the Nox4 protein, which leads to a decrease in the levels of reactive oxygen species and consequently, the release of the pro-inflammatory peptide (Basu et al., 2019).

## Cytotoxic/tumor activity

Some species of the *Euphorbia* genus exhibit antitumor activity against different cancer cell lines. The anticancer activity of the phenolic extract of *E. tirucalli* was evaluated in vitro on five cancer cell lines: MCF-7, A549, HL-60, HCT116, and HepG2. The IC_50_ values of the extract against the MCF-7 and A549 cancer cell lines were 1.65 ± 3.67 and 35.36 ± 3.82 μg/ml, respectively. In addition, it exhibited a potent cytotoxic activity against HL-60, with an IC_50_ value of 22.76 ± 2.85 μg/ml, while the IC_50_ value of doxorubicin was 21.87 ± 2.31 μg/ml. However, it had no activity against HepG2 and HCT116 cancer cells. These data suggest that these cancer cells were strongly affected by the phenolic compounds detected in the latex extract (Abdel-Aty et al., 2019). In another study of the same species; the crude latex extract of *E. tirucalli* reduced the viability of gastric adenocarcinoma cancer cells at concentrations of 100 and 200 μg/mL by up to 70 and 95%, respectively. This effect could be associated with euphol, which is the main compound found in this species (de Souza et al., 2019).

**FIGURE 13:**
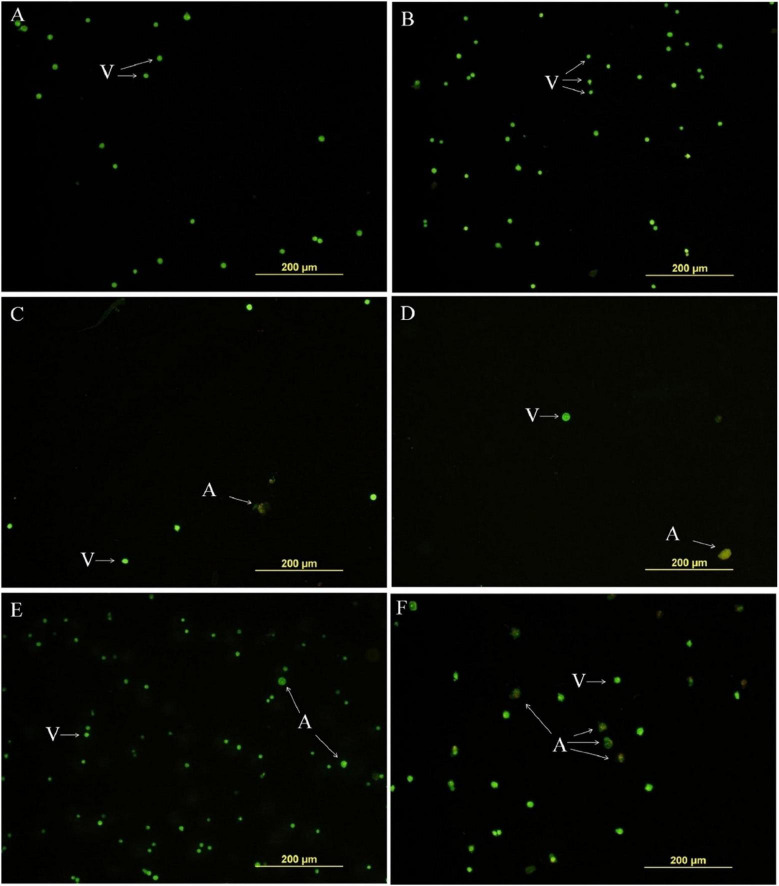
Euphol isolated from the latex of *E. tirucalli* inhibited total number of tumor cell K-562 cells after treatment (12 h). **(A)** Control cells incubated with RPMI only; **(B)** control cells incubated with DMSO (0.4%); **(C)** euphol treatment (23.4 μM); **(D)** euphol treatment (46.9 μM); **(E)** imatinib (0.5 μM); and **(F)** doxorubicin (0.085 μM). v, viable cells; a, apoptotic cells (Cruz et al., 2018).

**FIGURE 14:**
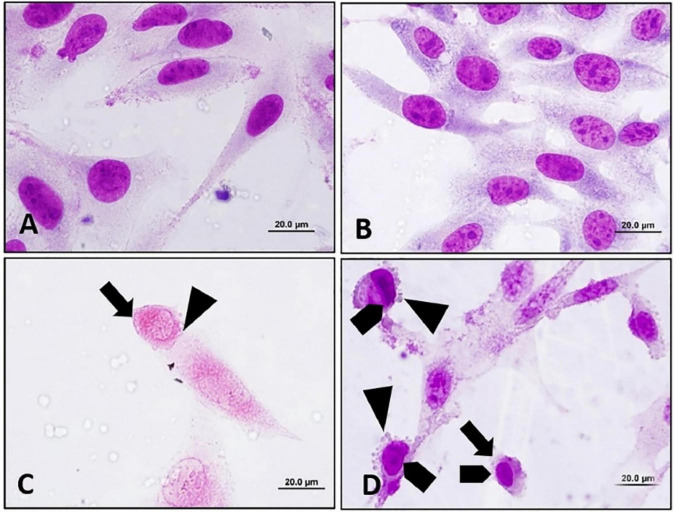
Apoptosis inducing effect of euphol extracted from *E. tirucalli* was to B16F10 cells after 24 h of treatment with different concentrations of euphol. **(A)** Control cells incubated with RPMI; **(B)** control cells incubated with DMSO (0.4%); **(C,D)** B16F10 cells treated with 70.3 and 35.2 μM of euphol. Cell rounding:, bleb formation:, chromatin condensation:. Magnification = 1,000×, bar = 20 μm (Cruz et al., 2018).

Cruz et al. investigated the cytotoxic effects of euphol isolated from the latex of *E. tirucalli* against the K-562 and B16F10 cell lines using the MTT assay and morphological analysis. It was observed that this compound shows high activity against both cell lines, with IC50 values of 34.56 ± 2.12 (μM) and 53.63 ± 10.16 (μM) after 72 h against K-562 and B16F10 cells, respectively. Similarly, morphological analysis of K-562 cells showed that, compared with the negative control (DMSO treatment) and the positive control (treatment with 0.085 μM doxorubicin and 0.5 μM imatinib), the group treated with 23.4 and 46.9 μM euphol had reduced total cell counts and contained apoptotic cells, as shown in Figure 13. In addition, morphological analysis of B16F10 cells after 24 h of treatment showed that euphol induces cell death through apoptosis accompanied by cell rounding, membrane bleeding, and chromatin condensation (Figure 14; Cruz et al., 2018).

**FIGURE 15:**
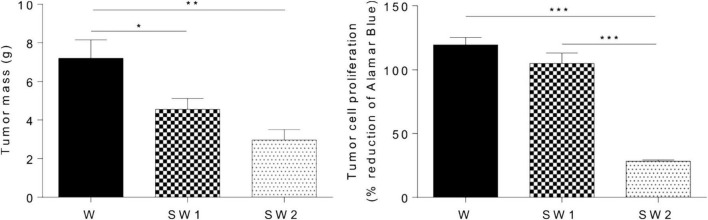
Effect of latex treatment on tumor mass and tumor cell proliferation in untreated animals with tumor (W), animals with tumor treated with 25 μL/mL aqueous solution of latex (SW1), and animals with tumor treated with 50 μL/mL aqueous solution of latex at (SW2). Data are presented as the mean + SEM. W, *n* = 11; SW1, *n* = 12; SW2, *n* = 14. *p < 0.05, **p < 0.001, and ***p < 0.0001 (one-way ANOVA followed by a post-hoc Tukey test) (Martins et al., 2020).

Subsequent work has recently shown that when the aqueous solution of latex from *E. tirucalli*, which contains triterpenes, is orally administered to male Wistar rats for 15 days, the tumor mass in the groups of rats treated with 25 μL latex/mL and 50 μL latex/mL latex is significantly lower than that in the control. Furthermore, a reduction of approximately 76% in tumor cell proliferation is observed in Wistar rats treated with 50 μl latex/ml (*p* < 0.0001), as determined by the Alamar Blue assay (Figure 15; Martins et al., 2020).

**FIGURE 16:**
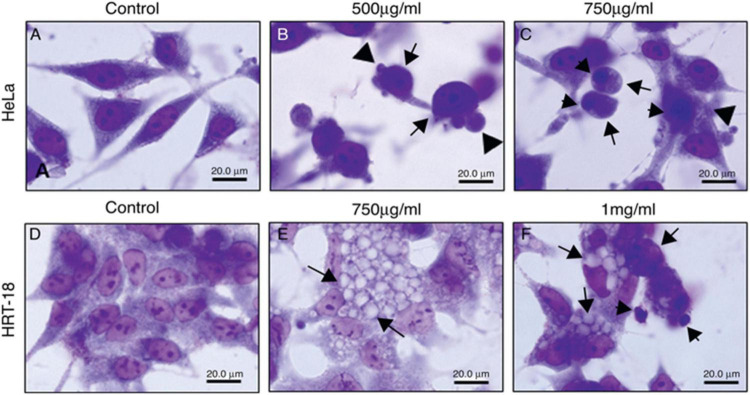
Morphological changes of HeLa and HRT-18 cells under treatment with latex of *E. umbellata* (Pax) Bruyn **(A,D)** controls; cells incubated with RPMI only. **(B)** HeLa cells incubated with 500 and **(C)** 750 μg/ml of latex. **(E)** HRT cells incubated with 750 μg/ml and **(F)** 1,000 μg/ml of latex (Luz et al., 2015).

Additionally, the cytotoxic activity of *E. umbellata* latex was tested by Luz et al. This study evaluated latex cytotoxicity on human cervical adenocarcinoma (HeLa) and human ileocecal colorectal adenocarcinoma (HRT-18) cells using the 3-(4,5-Dimethylthiazol-2-yl)-2,5-diphenyltetrazolium bromide (MTT) test and neutral red. The cell viability of HRT-18 cells was reduced after 48 h when 100 to 1,000 g/ml concentrations were used. Moreover, the latex induced dose- and time-dependent cytotoxicity to HeLa cells. A photomicroscope was used to analyze the cytotoxic effects of *E. umbellata* latex on HeLa and HRT-18 cell morphology, including vacuolization, rounding, loss of adhesion, blebbing, nuclear condensation, and fragmentation. After 24 h, morphological alterations in HeLa and HRT-18 cells were observed and were characterized by the loss of adhesion, cellular rounding, formation of bubbles, and condensation of chromatin, showing that apoptosis is the pathway for destruction tumor (Figure 16; Luz et al., 2015).

Another study was carried out to determine the concentration at which the latex extract of *E. antiquorum* exhibits maximum protection and least toxicity to cells. It has been reported to be safe to normal cells such as those of *brineshrimps* (*Artemia*), *S. cerevisiae*, and chick embryo fibroblast cells, and that the toxicity of latex increases with increasing concentration.

## Angiogenic and genotoxic/mutagenic activity

**FIGURE 17:**
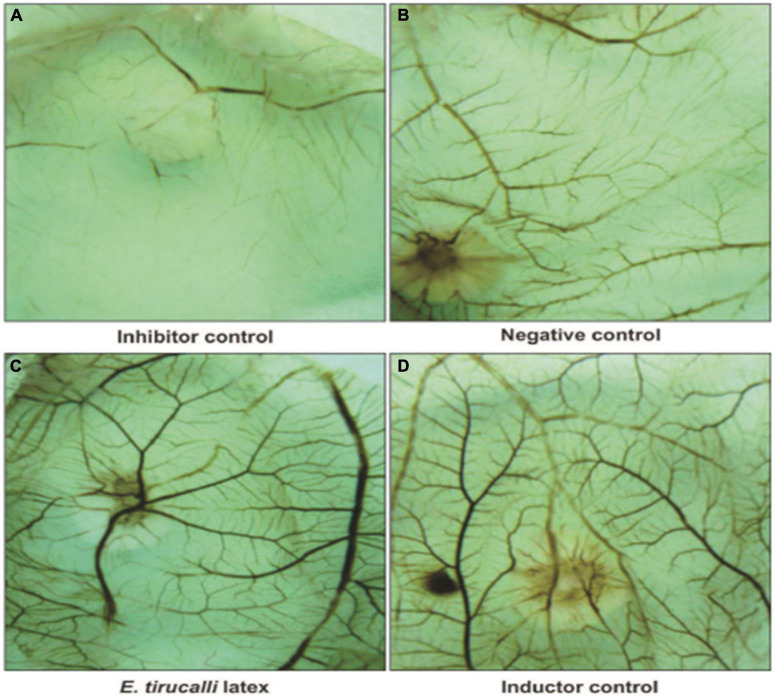
Photomicrography of Chorioallantoic membrane (CAM) vascular network formation. **(A)** Inhibitor dexamethasone; **(B)** the negative control (water); **(C)** the test solution (*E. tirucalli*); and **(D)** the inducer control (Biocure Biomembrane) (Bessa et al., 2015).

**FIGURE 18:**
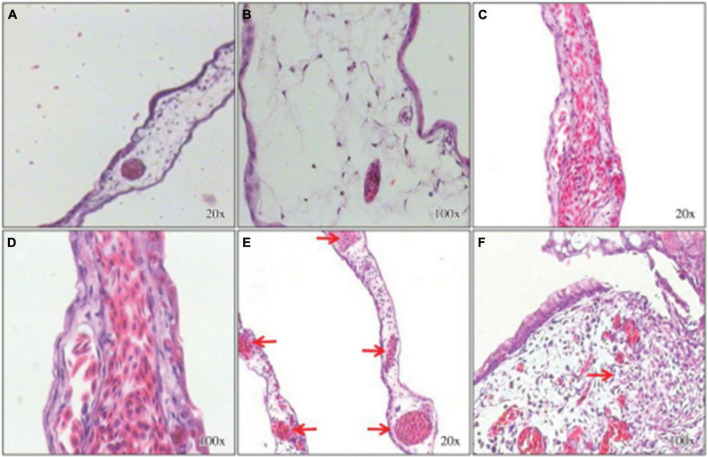
Histological sections stained with hematoxylin-eosin. Chorioallantoic membranes (CAMs) treated with the inhibitor control (dexamethasone) show few connective tissue cells and few blood vessels **(A,B)**. The inducer control (Biocure Biomembrane of Hevea brasiliensis latex) treatment induced a large number of blood vessels and inflammatory foci **(C,D)**. Treatment with the test solution of Euphorbia tirucalli latex resulted in a large number of well-organized blood vessels and inflammatory foci **(E,F)** (Bessa et al., 2015).

Angiogenesis is the growth of new vessels from an existing vascular system (Fan et al., 2006). In 2015, the pro-angiogenic activity of an aqueous *E. tirucalli* latex solution (10 mg/mL) was evaluated on chorioallantoic membranes (CAMs) of 80 fertilized chicken eggs through the application of a series of tests such as the quantification of the percentage of vascularization, histological analysis, and digital imaging; the aqueous solution significantly increased neoangiogenesis (CAM vascular network mean area and standard deviation of 46.3 ± 3.8 in the treated group versus 31.8 ± 3.0 in the control group). On the other hand, the mean surface of the vascular network in the inducer control group (51.3 ± 3.9) was not significantly (*p* > 0.05) different from that in the group treated with the *E. tirucalli* latex test. The digital images of the vascular networks of the control and the group treated with the aqueous solution of *E. tirucalli* latex are shown in Figure 17. The results of the histological analysis agreed with the results observed on the digital images (Figure 18). The positive control and *E. tirucalli* latex groups showed an increase in the number of blood vessels and an inflammatory response, whereas few blood vessels were found in the control group treated with 1% dexamethasone. Thus, the latex of *E. tirucalli* led to the activation of the inflammatory response (Bessa et al., 2015). On the other hand, the anti-angiogenic activity of *E. helioscopia* latex (100 μg/mL) has been studied in fertilized white leghorn hen eggs. The branching of blood vessels in the latex-treated groups was similar to that in the quercetin-treated group (standard). The genotoxic and mutagenic effects of *E. helioscopia* latex at different concentrations (1,000, 200, 40, 8, and 1.6 μg/ml) was evaluated by Saleem et al. No DNA damage was observed in the lymphocytes and *S. typhimurium* reverts in latex-treated plates could not be produce at any of the doses tested (Saleem et al., 2015b).

## Hemostatic and wound healing activity

Evaluation of various proteolytic activities such as protease, gelatinase, milk coagulation, and whole blood coagulation in the latex enzymatic fraction of *E. nivulia* Buch.-Ham revealed that this latex has hemostatic activity (Badgujar, 2014). Regarding proteolytic activity, the latex showed significant milk clotting activity with a value of 465.5 ± 0.37 U/g latex and protease activity with a value of 9.20 ± 0.08 U/g latex. In the gelatinase assay, the latex showed a value of 7.34 ± 0.72 U/g latex. Moreover, latex proteases have been shown to exhibit coagulation activity. Whole blood clotting times in mouse blood, human blood, and other mammals’ blood samples such as those from *Capra hircus*, *Bos indicus*, *Bubalus bubalis*, and *Ovibos moschatus* were reduced by treatment with proteases present in *E. nivulia* Buch.-Ham latex. Other work has examined the wound healing activity of *E. caducifolia* latex in excision and incision wound model mice and study the effect of this latex extract on hydroxyproline and DNA content.

**FIGURE 19:**
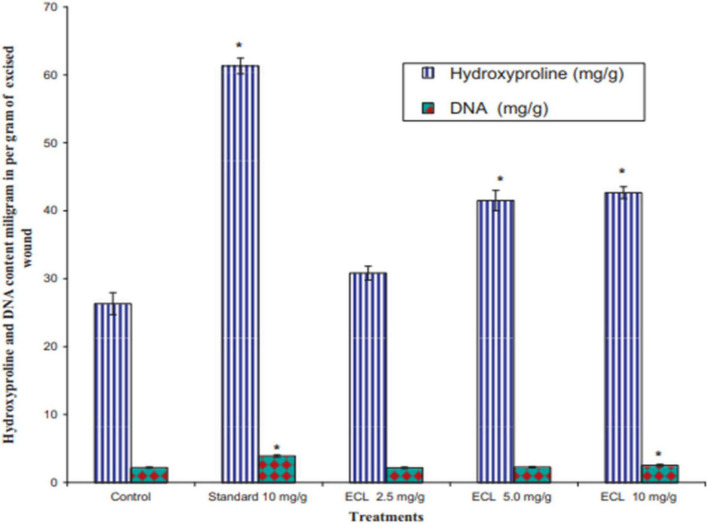
Effect of latex of *E. caducifolia* on hydroxyproline and DNA content. Values reported as Mean ± SEM (*n* = 6). The data were analyzed by one way ANOVA followed by and Dunnett’s test. *P0.05 as compared with control group. ECL (latex of *E. caducifolia*) (Goyal et al., 2012).

The results showed a complete closure of the wound in animals treated with *E. caducifolia* latex at concentrations of 2.5 and 5.0 mg/g after the 15th day. On the other hand, treatment with 10 mg/g allowed a total closure of the wound after the 14th day. Also the results of hydroxyproline content showed that the excised skin of animals treated with the latex extract with a concentration of 0.50 and 1.0 mg/g was found to have a higher amount of hydroxyproline compared to the control group, however the increase in DNA content was statistically significant only in the group treated with 10 mg/g ECL as shown in Figure 19.

In addition, the histopathological examination of excised skin showed the formation of new vessels with scattered inflammatory cells in mice treated with the latex of *E. caducifolia* (Goyal et al., 2012).

## Conclusion

To our knowledge, this review represents the first report summarizing the phytochemical analysis of spurge genus latex and its pharmacological effects. *Euphorbia* is one of the largest genera in the *Euphorbiaceae* family. This review summarizes the available literature to identify compounds with pharmacological activities extracted from the latex of different species of *Euphorbia*. The major constituent secondary metabolites of *Euphorbia* species are terpenoids, and most of them have been identified using HPLC, GC-MS, and NMR spectroscopic analysis. Latex extracts from *Euphorbia* species have many pharmacological functions, including antimicrobial, anticancer, anticholinesterase, anti-inflammatory, antioxidant, cytotoxic, anti-angiogenic, genotoxic/mutagenic, and wound healing activities, which have been demonstrated in various *in vitro* and *in vivo* biological test models. However, other components such as phenolic compounds, alkaloids, saponins, and flavonoids isolated from the latex of these species have been mostly ignored, which limits the diversity of application of the latex from these plants. This review summarizes the current understanding of the biological activities of secondary metabolites from the latex of *Euphorbia* species. Our findings may promote future studies that will help to optimize the therapeutic use of latex extracts and could be useful for scientists who need unexplored species that have not yet been fully explored.

However, few studies have tested the biological activities of the latex of the genus Euphorbia in vivo conditions, further investigations are recommended in order to better understand and discover more bioactive molecules. In addition, great attention should be paid to study the pharmacokinetics and the mechanism of action of the various compounds isolated from the latex of this genus.

## Author contributions

RB, AM, and AE: writing—original draft. NK and EC: writing—review and editing. NKK, NK, EC, and AE: funding and supervision. All authors contributed to the article and approved the submitted version.

## Abbreviations

ABTS: 2,2′-azino-bis( 3-ethylbenzothiazoline-6- sulphonic acid)
DPPH: 2,2-diphenyl-2-picrylhydrazyl
E: *Euphorbia*
FRAP: ferric reducing antioxidant power
FTIR: Fourier-transform infrared spectroscopy
GAE: gallic acid equivalents
IC50: 50% inhibitory concentration
LC50, lethal concentration: 50 percent
MIC: minimum inhibitory concentration
MTT: (3-(4,5-dimethylthiazol-2-yl)-2,5-diphenyltetrazolium bromide)
NMR: nuclear magnetic resonance spectroscopy
NO: nitric oxide
TEAC: trolox equivalent antioxidant capacity.
