# Supplementary material for: Euphorbia species latex: A comprehensive review on phytochemistry and biological activities
Source: Front Plant Sci. 2022 Oct 6;13:1008881. doi: 10.3389/fpls.2022.1008881 (PMC9583255; doi:10.3389/fpls.2022.1008881)
Supplement: Supplementary file 1 [file Table_2.docx]

**Table 2.** Summary of the biological activities for euphorbia genus latex

| **Activity** | **Species** | **Study design** | **Mode** | **Fractions** | **Main finding** | **References** |
| --- | --- | --- | --- | --- | --- | --- |
| **Antibacterial** | ***E. hirta*** | antibacterial activity was evaluated by the disc diffusion method against: Bacillus pumilus, *Staphylococcus aureus, Streptococcus pneumoniae, Escherichia coli, Citrobacter freundii, and Klebsiella pneumoniae,* | In- vitro | Fresh latex & Diluted latex | Fresh latex of *E. hirta* inhibited *B. pumilus* with an inhibition zone of 24.98 mm, *S. aureus* (25.38mm), *S. pneumoniae* (23.72mm), *E. coli* (27.93mm), *C. freundii* (23.54mm) and *K. pneumoniae* (21.93mm), with relative percentages of inhibition 125.63, 106.10, 111.15, 129.42, 110.82, and 102.16 respectively -The diluted latex (1: 10) in water of the *E. hirta* showed no antimicrobial response. | Hussain et al., 2014 |
|  | ***E.thymifolia*** | antibacterial activity was evaluated by the disc diffusion method against: *B. pumilus, S. aureus, S.pneumoniae, Escherichia coli, C. freundii, and K.pneumoniae* | In- vitro | Fresh latex & Diluted latex | Fresh latex of *E. thymifolia* l showed inhibitory activity against *B. pumilus* (20.37mm), *S. aureus* (22.82 mm), *S. pneumoniae* (20.94mm), *Escherichia coli* (23.74mm), *C. freundii* (18.82mm) and *K. pneumoniae* (18.32mm). -The diluted latex (1: 10) in water of the *E. thymifolia* showed no antimicrobial response. | Hussain et al., 2014 |
|  | ***E. antiquorum*** | The antibacterial activity was carried out by the disc diffusion and the Agar well diffusion method against: *K. pneumoniae, Shigella flexneri, S. aureus, Bacillus subtilis and E.coli.* | In- vitro | Methanolic extract of latex | The results obtained for disc diffusion was similar to the Agar well diffusion method. Latex extract (1ml of latex in 5 ml of methanol) inhibited *E. coli* with an inhibition diameter of 5mm and *S. flexneri* with an inhibition diameter of 4mm; whereas did not show an inhibition against *S*. *aureus, K. pneumoniae* and *B. subtilis.* | Sumathi et al., 2011 |

**Table 2. (Continued)**

| **Activity** | **Species** | **Study design** | **Mode** | **Fractions** | **Main finding** | **References** |
| --- | --- | --- | --- | --- | --- | --- |
| **Antibacterial** | ***E. caducifolia*** | Anti-bacterial activity of latex and isolated fraction was tested by a modified well-in agar method and by broth microdilution method | In- vitro | -Latex and fraction isolated from latex: 3-Oxo-25, 26, 27-trisnor (5a, 13a,14b,17a) lanost-8-en-24-al, methyl palmitate, 5,9- heptadecadienoate, methyl 11 octadecenoate, methyl octadecenoate, 3,7,11,15-tetramethyl-2 hexadecene-lol (Phytol) and lanost-8-en-24-al. | The isolated fracture of *E.* The isolated fracture of *E. caducifolia* (IFEC) at 500 µg / ml showed good antibacterial activity compared to latex. the (IFECs) marked an inhibition diameter of 16.2 ± 0.22mm against *S. aureus*, 14.4 ± 0.33m against *Micrococcus luteus*, 14.5 ± 0.26 mm against *B. subtilis*, 15.1 ± 0.41mm against *E. coli* and 16.4 ± 0.51mm against *Salmonella typhi*. On the other hand, the latex of *E. caducifolia* showed (9.3 ± 0.21 mm zone of inhibition) against *S. aureus*, (9.2 ± 0.31mm zone of inhibition) against *M. luteus*, (8.1 ± 0.25mm zone of inhibition) against *B. subtilis*, (9.4 ± 0.24mm zone of inhibition) against *E. coli*  and (8.2 ± 0.33mm zone of inhibition) against *S. typhi.*  The minimum inhibitory concentrations of the isolated fraction of *E. caducifolia* (IFEC) are 262µg, 212µg, 187µg, 225µg and 275µg against *S. aureus,* *M luteus*; *B. subtilis,* *E. coli* and *S. typhi* respectively. For latex, the MICs are 458 µg, 450 µg, 475 µg, 525 µg and 500 µg against *S. aureus, M. luteus; B.*  subtilis, *E. coli* and *S. typhi .*respectively. | Goyal et al., 1970 |

**Table 2. (Continued)**

| **Activity** | **Species** | **Study design** | **Mode** | **Fractions** | **Main finding** | **References** |
| --- | --- | --- | --- | --- | --- | --- |
| **Antibacterial** | ***E. officinarum*** | The in vitro antibacterial activity of triterpene derivatives of the latex of *E. officinarum* against the following phytopathogens: *Pseudomonas syringae pv. syringae, P. syringae pv. tabacci, Erwinia amylovora* and *Agrobacterium tumefaciens* using disc diffusion method. | In- vitro | 3β-acetoxy-norlup-20-one and 3-chloro 4α, 14α-dimethyl-5α-cholest-8-ene. | The effect of derivatives on spore germination of fungal phytopathogens showed that the IC_50_ of both compounds was even lower than that of methyl thiophanate for 5 strains of *Verticillium dahliae*.  The effect of mycelial growth, revealed that the inhibition of the two compounds tested were significantly lower (IC50 >500µg/ml) than those of methyl thiophanate (IC50 <2µg/ml) fungal phytopathogens. The formation of conidia was significantly reduced compared to the control by Compounds 1 and 2 from 39 to 96% for the strains *V. dahliae*, *Penicillium expansum* and *Fusarium oxysporum.*  3-chloro 4α, 14α-dimethyl-5α-cholest-8-ene showed a good activity similar to chloramphenicol (16mm zone of inhibition) and (6mm zone of inhibition) against *P. syringae pv. Syringae and syringae pv. Tabaci* respectively*.* 3-chloro 4α, 14α-dimethyl-5α-cholest-8-ene also induced a moderate antibacterial activity at 100 µg mL^-1^against *E. amylovora*. | Smaili et al., 2017 |

**Table 2. (Continued)**

| **Activity** | **Species** | **Study design** | **Mode** | **Fractions** | **Main finding** | **References** |
| --- | --- | --- | --- | --- | --- | --- |
| **Antibacterial** | ***E.heterophylla*** | The in vitro antibacterial activity was tested by disk diffusion method against *S. aureus* (MTCC 7443), *B. subtilis* (MTCC 121), *P. vulgaris*, and *Pseudomonas aeruginosa* (NCIM 2200) | In- vitro | acetone, chloroform, and diethyl ether extracts | All extract showed antibacterial activity against these bacteria at different concentrations, acetone extract showed a high potential against *P.* aeruginosa (14 mm at 15% concentration), diethyl ether extract showed a high potential against *P. vulgaris* (with an inhibition zone of 15 mm at the 15% concentration) and Chloroform latex extract and showed a high antibacterial activity against *S. aureus* and *B. subtilis* (with 9 mm at the 15% concentration) | ML et al., 2020 |
|  | ***E. abyssinica*** | The in vitro antibacterial activity was estimated by the agar disk diffusion assay against *S. aureus*, *E. Coli*, *S. typhi* and *P. aeroguinosa* | In- vitro | Absolute methanol, Aqueous and  50% Methanol  extracts | The absolute and 50% methanol extracts (100mg/ml) showed an antibacterial activity against S. aureus with an Inhibition zone diameters 13±0.82 mm, 15±1.60 respectively and against *P. aureoguinosa* 15± 0.82 mm and 17±1.16 mm respectively. However, a similar antibacterial activity against *E. coli* (18 ±1.60 mm), *S. typhi* (17 ± 0.82 mm) and respectively.  The aqueous extract (100 mg/ ml) showed showed an intermediate activity: 12 ± 0.8mm, 10 ± 1.60 mm, 11 ± 0.08mm and 11 ± 1.16 mm against *E. coli*, *S. aureus*, *S. typhi* and *P. aeruginosa* respectively. | Tarh and Iroegbu, 2019 |

**Table 2. (Continued)**

| **Activity** | **Species** | **Study design** | **Mode** | **Fractions** | **Main finding** | **References** |
| --- | --- | --- | --- | --- | --- | --- |
| **Antioxidant** | ***E. Tirucalli*** | The antioxidant activities of the *E.* *tirucalli* latex extracts were determined in vitro by DPPH, ABTS and Phosphomolybdenum complex assay. | In- vitro | Methanol latex extract | The latex extract contains phenols which possessed powerful antioxidant activity.  The antioxidant activity of the phenolic content of the *E. tirucalli* latex extracts was evaluated on the basis of their ability to scavenge free radicals DPPH and ABTS, which had IC_50_ values of 6.0 µg GAE / ml and, 2.0 µg GAE / ml, respectively.  The total antioxidant activity, by the use of phosphomolybdate assay, of the latex extract of *E. tricullari* was also measured with EC_50_ values of 6.5 μg GAE / ml | Abdel-Aty et al., 2019 |
|  | ***E. dendroides L*** | The antioxidant activity was determined in vitro using free radical scavenging activity (DPPH assay), ferric reducing (FRAP), TEAC and ABTS assay | In- vitro | Méthanol extract | *E. dendroides* latex extract (0.625–10 µg/ mL) showed antioxidant activity. The extracts inhibited DPPH absorption with values (2927.01 ± 98.03 mmols of Trolox equivalent (TE)/100g FW). Reactivity towards ABTS radical cation and ferric-reducing antioxidant power (FRAP) values were 7580.95 ± 97.65 mmols of TE/100g FW and 4383.13 ± 95.30 µmol of TE/100g FW, respectively. | Smeriglio et al., 2019 |

**Table 2. (Continued)**

| **Activity** | **Species** | **Study design** | **Mode** | **Fractions** | **Main finding** | **References** |
| --- | --- | --- | --- | --- | --- | --- |
| **Antioxidant** | ***E. Bicolor*** | The antioxidant activities of *E. bicolor* latex extract were determined by Ferric reducing power, 2,2-Azino-bis-(3-ethylbenzothiazoline-6-sulphonic acid) (ABTS) scavenging activity, Free radical scavenging activity (DPPH), Hydrogen peroxide scavenging activity and Nitric oxide (NO) scavenging activity | In- vitro | *E. bicolor* latex extract | The IC50 values of the latex extract required to extinguish 50% of the free radicals are: (18 2 ± 13 μg / mL) for the ABTS radical test, (68 4 ± 3 8 μg / m) L for the DPPH radical , (50 ± 6 6 μg / mL) for raducal the H2O2 radical and (1 6 ± 0 04 μg / mL) for nitric oxide radical | Basu et al., 2019 |
| **cytotoxic activity** | ***E. Tirucalli*** | The cytotoxic activity of the phanolic content of *E. Tirucali* methanolic latex extract against Acute myeloid leukemia (HL-60), breast MCF-7, liver HepG2, colon HCT116 and lung A549 cancer cell lines at (12.5–100 μg GAE/ml) in vitro ; using SulfoRhodamine-B stain (SRB) assay | In- vitro | methanol latex extract | The phenolic latex extract of *E. tirucali* showed a potent cytotoxic activity in the, MCF7, cancer cell lines; with IC50 value 31.65 ± 3.67 μg/ ml compared to o the reference drug, doxorubicin with IC50 values of 24.50 ± 1.72 μg/ml. Also found to be active against A549 and HL-6 with 35.36 ± 3.82 and 22.76 ± 2.85a μg/ml compared to the doxorubicin with IC50 values of 23.84 ± 2.43 and 21.87 ± 2.31 μg/ml, respectively. Furthermore, the latex extract did not exert any activity against HepG2 and HCT116 cancer cells. | Abdel-Aty et al., 2019 |

**Table 2. (Continued)**

| **Activity** | **Species** | **Study design** | **Mode** | **Fractions** | **Main finding** | **References** |
| --- | --- | --- | --- | --- | --- | --- |
|  | ***E. tirucalli*** | The cytotoxic activity of crud extract was investigated with various concentrations (3.125–200 μg/mL) against macrophages RAW 264.7 and Gastric Adenocarcinoma (AGS) cancer cells using MTT assay. | In- vitro | The crude latex | The crude latex tested showed no significant toxic effect against RAW 264.7 macrophages. At 215.14 ± 0, 28 μg/mL, there was a viability reduction of only 10%. The IC50 values for *E. tirucalli* is 521.60 ± 0.01 μg/mL compared to reference cytotoxic agent with IC50 values 4.40 ± 0.53 μg/mL. Furthermore, the latex at the concentration of 200 μg/mL reduced the viability of the AGS cells in 95%, high cytotoxicity of crude llatex (69.43 ± 1.29 μg/mL) against gastric adenocarcinoma tumor cells and 4.35 ± 0.9 μg/ mL for cisplatin | de Souza et al., 2019 |
| **Cytotoxic/tumor activity** | [***E.***](https://www.sciencedirect.com/topics/agricultural-and-biological-sciences/euphorbia)***umbellata*** | The cytotoxic activity of E. umbellata latex was tested on different tumor cell lines: Human cervical adenocarcinoma – HeLa (ATCC CCL-2) at 500 and 750 μg/ml and human ileocecal colorectal adenocarcinoma – HRT-18 (ATCC CCL-244) at 750 and 1000 μg/ml cells by y employing MTT and Neutral Red (NR) techniques. | In- vitro | The latex | The phenolic latex extract of *E. tirucali* showed a potent cytotoxic activity in the, MCF7, cancer cell lines; with IC50 value 31.65 ± 3.67 μg/ ml compared to o the reference drug, doxorubicin with IC50 values of 24.50 ± 1.72 μg/ml. Also found to be active against A549 and HL-6 with 35.36 ± 3.82 and 22.76 ± 2.85a μg/ml compared to the doxorubicin with IC50 values of 23.84 ± 2.43 and 21.87 ± 2.31 μg/ml, respectively. Furthermore, the latex extract did not exert any activity against HepG2 and HCT116 cancer cells. | Luz et al., 2015 |

**Table 2. (Continued)**

| **Activity** | **Species** | **Study design** | **Mode** | **Fractions** | **Main finding** | **References** |
| --- | --- | --- | --- | --- | --- | --- |
|  | ***E. antiquorum*** | MTT assay, SRB assay, Neutral red assay and Ethidium bromide staining (EtBr) were used to determine the cytotoxic activity of methanolic extract of different concentrations of E. antiquorum latex (10, 20, 30, 40 and 50 µg) in different models: S. cerevisiae cells, shrimps and chick embryo fibroblast cells. | In Vitro | Methanolic latex extract | The latex extract exhibited minimal cytotoxic effects on *S. cerevisiae* cells in a concentration-dependent manner. At the lowest concentration, shrimp survival rate was increased with LC50 = 6.76 µg ml-1 after 24 hours. The latex extract does not induce apoptosis in normal cells but modulates the apoptotic effects produced by etoposide in chicken embryo fibroblast cells. | Sumathi et al., 2011 |
| **Cytotoxic/ tumor activity** | ***E. tirucali*** | The tumor cell proliferation of aqueous latex solution of *E. tirucalli* was tested in Male Wistar rats (∼350g).The rats were received 1 mL of latex solution (25µl/ml and 50µl/ml) and after 15 days the tumor mass was determined and the proliferative capacity of tumor cells were evaluated using the Alamar Blue assay. | In Vitro | aqueous solution of latex | The group treated with 50 μL/mL of latex showed a 76% reduction in the proliferation of tumor cells compared to the control. While the tumor mass was reduced by 40% and 60% in the groups treated with 25µg/ml and in the animals treated with 50µL/ml of latex respectively. | Martins et al., 2020 |

**Table 2. (Continued)**

| **Activity** | **Species** | **Study design** | **Mode** | **Fractions** | **Main finding** | **References** |
| --- | --- | --- | --- | --- | --- | --- |
|  | ***E. royleana*** | Charles Foster rats male (120-160g) and Swiss albino mice (18-24g) were used to testing anti-inflammatory and anti-arthritic effects. Hydrosoluble fraction of *E. royleana* latex (50 to 200mg/kg) was given 45 min before noxious agents injection. Acetylsalic acid (50-100mg/kg) was used as a standard drug. | In vitro | hydrosoluble fraction | The hydrosoluble fractoion caused a significant reducing of the oedema induced by carrageenan and dextran in rats. Formaldehyde induced arthritis was significantly reduced in a dose dependent (the effect was in its maximum (35%). Carrageenan-induced pleurisy in rats; the hydrosoluble fraction was significantly reduced in a dose dependent the exudate volume and total leukocyte count in the pleural cavity. | Bani et al., 2000 |
| **Anti-Inflammatory** | ***E. bicolor*** | The anti-inflammatory effect of latex extract was tested in male and female adult Sprague-Dawley rats (250-350 g). The rats were injected by 50 μL complete Freund’s adjuvant into the left vibrissal pad to induce inflammation and then injected by *E. bicolor* latex extract (300 μg/mg in 0.9% saline and <5% methanol. | In-Vivo | Methanolic latex extract | The latex extract of E. bicolor reduced orofacial mechanical sensitivity in male rats at 24 hours and in female rats at 72 hours. In female rats, latex extract significantly down-regulated different pro-inflammatory cytokines (IL-1β, IL-2, and IL-3) and pro-inflammatory chemokines (MIG) in trigeminal ganglia after 1 hour, and in male rats, it significantly down-regulated some pro-inflammatory cytokines (IL-1α, IL-1β, IL-2, IL-3, and IL-17), chemotactic cytokines (MIP-1α and MIP-3α), and pro-inflammatory chemokines (CINC-1, MIG) and upregulated an anti-inflammatory chemokine (TIMP) in trigeminal ganglia after 1 hour | Basu et al., 2019 |

**Table 2. (Continued)**

| **Activity** | **Species** | **Study design** | **Mode** | **Fractions** | **Main finding** | **References** |
| --- | --- | --- | --- | --- | --- | --- |
| **Anti-Inflammatory** | ***E. helioscopia*** | Albino mice with either sex weighing 25-30 g were used to testing anti-inflammatory activity of the latex. The sample was given at dose levels 100, 200 and 300 mg/kg and the Carrageenan induced paw edema was used by injection of Carrageenan (1%, 0.05 mL) in the plantar surface of right hind paw of individual mouse half an hour after administration. | In vitro | The latex | The latex caused a significant reduction in the volume of the rat paw (P < 0.05). The maximum anti-inflammatory activity was 59.38% at concentrations of 200 mg/kg. | Saleem et al., 2015a |
| **Wound healing activity** | ***E. caducifolia*** | Albino rats both sex weighting (150-170g) were used in this study to evaluate the wound healing effect of *E. caducifolia* latex by incision wound model.  However mice weighting (24-28g), were used for excision wound model. | In vivo | Ointment of latex (2.5, 5.0 and 10 mg/g) | The excised skin treated with a dose of 10 mg / g contained the highest amount of hydroxyproline and DNA, thus it showed 100% contraction after 14 days after wounding.  The skin excised from rats treated with extract of latex at 10 mg / g was found to have the highest tensile strength with a value of 511.83 ± 17.67 g / cm^2^ (P <0.05). | Goyal et al., 2012 |

**Table 2. (Continued)**

| **Activity** | **Species** | **Study design** | **Mode** | **Fractions** | **Main finding** | **References** |
| --- | --- | --- | --- | --- | --- | --- |
| **Hemostatic activity** | ***E. nivulia Buch.-Ham*** | The latex of *E. novilia Buch.-Ham* species was tested on fresh sections to stop bleeding in Swiss albino mice, by inducing a sting in the tail of the mice and applying a drop of two different doses to the crude enzyme extract of the latex (protein content of 50 and 100 μg).  Latex was also applied to wounds to study its effect to enhance the healing process. | In- Vivo | latex crude enzyme extract | The protein fraction of *E. novilia Buch.-Ham* latex significantly reduced the bleeding time of mice from 57.17 ± 0.98 to 26.83 ± 1.16 s (P <0.05) at a dose of 100 μg (Protein content), compared to the positive and negative control group of mice. | Badgujar, 2014 |
| **Anti-Angiogenic Activity** | ***E. helioscopia*** | The fertile white leghorn chicken eggs were used to evaluate the anti-angiogenic activity of *E. helioscopia* by CAM assay, through the application of 200 µL, containing 10 µg/mL, 30 µg/mL, 50 µg/mL, 80 µg/mL, and 100 µg of concentration of sample/ 100 µg/mL of standard on the growing embryo. | In- Vivo | Latex obtained by cutting the leafy part from the stems. | The latex showed specific angiogenic activity. It showed a decrease in branching of secondary and tertiary blood vessels and a decrease in their thickness.  The latex showed a non-significant difference in the anti-angiogenic property of 10.50%. | Saleem et al., 2015b |

**Table 2. (Continued)**

| **Activity** | **Species** | **Study design** | **Mode** | **Fractions** | **Main finding** | **References** |
| --- | --- | --- | --- | --- | --- | --- |
| **Anti-Angiogenic Activity** | ***E. tirucalli*** | The pro-angiogenic activity of aqueous *E. tirucalli* latex solution (10 mg/mL) determined by the chorioallantoic membrane assay of 80 fertilized eggs obtained from Rhoss breed chicken (Gallus domesticus). | In- Vivo | The aqueous latex solution | The data reveled that aqueous latex solution (10 mg/mL) significatenly increased vascular network formation (p < 0.05); with a mean area and standard deviation of 46.3 ± 3.8, comparing to the negative control which showed mean area and standard deviation of 31.8 ± 3.0. In contrast, the inducing control group (51.3 ± 3.9) was not significantly different from that of the *E. tirucalli* latex test group (p> 0.05). | Bessa et al., 2015 |
| **Genotoxic /mutagenic activity** | ***E. helioscopia*** | The latex has been tested at different concentrations (1000, 200, 40, 8 and 1.6 µg/ml) to study its genotoxic potential using the Comete test and its mutagenic effect by the Maron and Ames test. | In- Vivo | Latex obtained by cutting the leafy part from the stems. | The latex did not show mutagenic or genotoxic activity, since it did not damage the DNA of lymphocytes and did not produce *S. typhimurium* agents even at a concentration of 1000 µg / ml. | Saleem et al., 2015c |

**Table 2. (Continued)**

| **Activity** | **Species** | **Study design** | **Mode** | **Fractions** | **Main finding** | **References** |
| --- | --- | --- | --- | --- | --- | --- |
| **Insecticidal activity** | ***E. antiquorum L*** | The Potters’ spray method, microapplicator method, leaf-dip method and hand-sprayer method, were used to determine the Insecticidal properties of *E. antiquorum* latex at differente concentrations : 0.05%, 0.025%, 0.0125%, 0.006% and 0.003% (in 20% newkalagen), against six insect pest species, two predatory Coccinellid species and a predatory spider. | In- Vivo | Dichloromethane, petroleum ether, n-hexane, acetone, xylene, methanol and distilled water latex extract | Xylene extraction showed better insecticidal components.  Only the 10% latex solvent extracts gave 100% mortality, except for the extract with distilled water. The xylene latex extract showed the highest activity. The three aphid species, A. craccivora, A. gossypii and M. persicae, showed a high level of mortality with an LC 50: 0.0089%, 0.0077%, 0.0119% respectively. | De Silva et al., 2008 |
